# Cholinergic Modulation of Proteinoid Microsphere Networks as Prebiotic Depression Models

**DOI:** 10.1021/acsphyschemau.6c00066

**Published:** 2026-06-13

**Authors:** Panagiotis Mougkogiannis, Andrew Adamatzky

**Affiliations:** Unconventional Computing Laboratory, 1981 University of the West of England, Coldharbour Lane, Bristol BS16 1QY, U.K.

**Keywords:** Proteinoid microspheres, cholinergic modulation, depression models, prebiotic chemistry, electrochemical oscillations

## Abstract

The molecular origins of mood disorders remain obscured by the overwhelming complexity of biological neural networks. Proteinoid microspheres are cell-like structures composed of amino acids, formed through heat-driven polymerization. Spontaneous electrical activity is observed in these microspheres and is modulated by nicotine, a potent cholinergic agonist associated with depression. Electrochemical characterization using impedance spectroscopy and cyclic voltammetry, combined with long-duration monitoring (over 75 h), demonstrates that nicotine induces a “depressive” state in prebiotic networks, defined here as a suppression of coherent, high-amplitude spiking activity. Crucially, this suppression is not a reduction in total activity: nicotine induces hyperactive but low-fidelity dynamics, in which increased firing frequency is accompanied by degraded signal organization and reduced informational content. This state is not defined by reduced activity, but rather by degraded signal quality. A 52% increase in firing frequency is accompanied by reduced amplitude precision, as deterministic dynamics collapse into stochastic noise. In parallel, phase-space volume expands by 1500-fold, indicating a major breakdown in homeostatic regulation. Equivalent circuit modeling shows that nicotine decreases membrane charge-transfer resistance by up to 90%, while capacitance increases 3-fold and exhibits chaotic fluctuations. These effects are consistent with membrane permeabilization and “shunting inhibition,” which suppress threshold depolarization. The transition is marked by a shift from self-organized criticality (fractal dimension *D* ≈ 5.0) to low-dimensional stochasticity (*D* ≈ 1.5). Shannon entropy increases by 1.67 bits, quantifying the thermodynamic cost of the depressed state as information leakage. These results indicate that cholinergic modulation of excitability is not solely a biological phenomenon dependent on evolved receptors, but rather a fundamental physicochemical interaction that may have influenced the emergence of nervous systems. It is proposed that depression reflects a breakdown in thermodynamic self-organization, in which an information-processing system shifts from ordered dynamics to chaotic disorder. These results point toward a physicochemical precursor to this principle, potentially extending its origins back to the prebiotic era—long before the evolutionary emergence of the first synapse.

## Graphical abstract

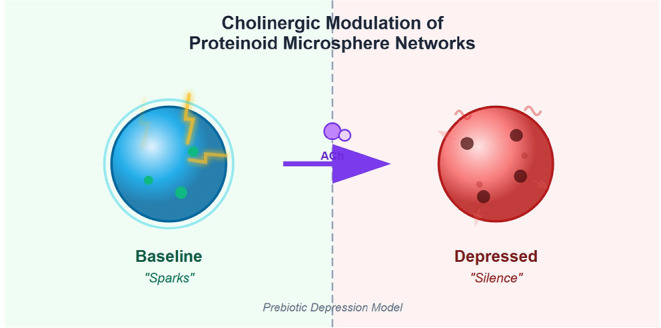

## Introduction

The origins of life and the emergence of neural computation represent two of biology’s most profound questions.^1,2^ Prebiotic chemical systems might have shown early signs of electrical excitability and information processing, suggesting that these capabilities could have emerged even before neurons evolved.^3,4^ Proteinoid microspheres, cell-like structures synthesized by the thermal polymerization of amino acids,^3^ serve as robust models for studying protocellular behavior. These abiotic assemblies spontaneously form semipermeable membranes, maintain electrochemical gradients, and exhibit electrical oscillations capable of mimicking neuronal action potentials.^5–10^

Recent advances in unconventional computing have characterized proteinoid networks as excitable media capable of spike propagation, Boolean logic operations, and basic memory formation.^4,11,12^ This excitability, grounded in fundamental chemical and physical structures, suggests that the mechanisms underlying neuronal function may be older and more universal than previously thought.^13,14^ If prebiotic systems could generate electrical signals, they might also have been susceptible to chemical modulation, potentially hinting at the precursors of neurotransmitter systems.^15,16^

Nicotine, a potent alkaloid and cholinergic agonist, acts as a powerful molecular probe for excitability.^17,18^ The cholinergic–adrenergic hypothesis, proposed by Janowsky et al., posits that depression arises from a dominance of cholinergic over adrenergic activity in the central nervous system.^19^ Under this framework, excessive stimulation by agonists like nicotine can mimic a depressive pathological state.^20,21^ Clinical observations support this: nicotinic stimulation can induce depressive symptoms in healthy individuals, while anticholinergic agents often alleviate them.^20,22^ However, the precise link between agonist-induced overactivity and depressive dynamics remains obscured by the biological complexity of in vivo systems.

We propose a bold simplification: the use of proteinoid microsphere networks as a prebiotic model to isolate the effects of nicotine on electrical activity. This approach offers distinct advantages. First, proteinoids provide a minimal substrate stripped of genomic and metabolic noise, allowing direct observation of molecular effects on electrical dynamics. Second, their spontaneous excitability provides a baseline “active” state against which pharmacological modulation can be measured. Third, as prebiotic analogs, they allow us to test if susceptibility to alkaloids is an archaic chemical interaction that predates the evolution of specific membrane receptors.^23,24^

It is important to note that nicotine was incorporated during thermal synthesis rather than applied postsynthetically to preformed microspheres. This protocol was chosen to produce a stable composite material, but it means that the observed effects likely reflect physicochemical modification of the proteinoid matrix rather than activation of evolved cholinergic receptor-like structures. The term “cholinergic-like” is therefore used here in a loose, analogical sense, referring to the functional similarity of the observed electrical suppression to cholinergic hyperactivity phenomenology, not to a mechanistic equivalence.

We demonstrate that nicotine exposure suppresses electrical spiking in proteinoid networks in a concentration-dependent manner, effectively creating a chemically induced “depressive” state. In the framework of this study, “depression” is used strictly as an operational dynamical analogy: it refers to the reduction of coherent, high-fidelity spiking activity and the suppression of membrane excitability, and does not imply a direct model of clinical or psychiatric depression, nor of cholinergic receptor-mediated neurotransmission.^25^

Table 1 shows the path that makes this hypothesis testable. It highlights three research areas that our work combines. The first lineage starts with Miller’s key study in 1953. He showed that amino acids, the building blocks of life, could form without life under early Earth conditions.^26^ Fox and colleagues expanded on this idea by demonstrating that heating these amino acids creates proteinoid microspheres. These microspheres are stable and resemble cells, with semipermeable membranes.^3,27^ These were not merely inert aggregates; crucially, Bachmann et al. demonstrated that such compartments could exhibit autocatalytic self-replication without genomic machinery,^28^ while Hanczyc et al. showed that even simpler lipid-based protocells could achieve chemotaxis and autonomous movement.^29^ Deamer et al. further showed that protocell membranes can keep ion gradients and create electrochemical potentials. This finding links prebiotic chemistry to the bioelectrical features of living cells. Ishima and Przybylski made an important discovery when they found that proteinoid microspheres can create membrane potentials and show electrical oscillations on their own.^5^ This ability to organize electrochemically was key to their finding. Recent work by Adamatzky and his team has detailed these oscillations. They showed that proteinoid networks act like excitable media capable of information processing. These networks can propagate spikes, perform Boolean logic, and carry out memory operations similar to neuronal computation.^4,11^

**Table 1:** Comparison of Foundational Studies Bridging Abiotic Protocells, Electrical Signaling, and Cholinergic Mechanisms^a^

Study	Focus Area	Key Findings and Relevance
Fox et al. (1958, 1995)	Origins of Life	Demonstrated thermal polymerization of amino acids into proteinoid microspheres, establishing them as stable, cell-like protocells with semipermeable membranes.^3,27^
Miller (1953)	Prebiotic Chemistry	Demonstrated abiotic synthesis of amino acids under simulated early Earth conditions, providing the molecular building blocks for proteinoid formation.^26^
Ishima & Przybylski (1981)	Biophysics	Early observation of spontaneous membrane potentials and electrical oscillations in proteinoid systems, suggesting primitive excitability.^5^
Bachmann et al. (1992)	Self-Replication	Demonstrated the autocatalytic self-replication of surfactant micelles, proving that simple chemical compartments can grow and replicate without genomic machinery.^28^
Loewi (1921)	Neuroscience	First demonstration of chemical neurotransmission (discovery of “Vagusstoff”, later confirmed as acetylcholine), establishing the basis for cholinergic signaling.^33^
Adamatzky et al. (2021–2026)	Unconventional Computing	Characterized proteinoids as electrically excitable media capable of Boolean logic, memory, and spike propagation similar to neuronal action potentials.^4,11^
Hodgkin & Huxley (1952)	Neurophysiology	Quantified the ionic mechanisms of action potentials, establishing mathematical frameworks applicable to any excitable membrane system including protocells.^30^
Janowsky et al. (1972)	Psychiatry	Proposed the cholinergic–adrenergic hypothesis of mania and depression, linking central cholinergic overactivity to depressive symptoms.^19^
Dilsaver (1986)	Psychopharmacology	Reviewed cholinergic mechanisms in affective illness, demonstrating that muscarinic receptor supersensitivity may contribute to depression vulnerability.^25^
Furey & Drevets (2006)	Clinical Neuroscience	Showed that anticholinergic agents produce rapid mood improvement in depressed patients, providing direct evidence for cholinergic involvement in depression.^34^
Deamer (2017)	Membrane Biophysics	Demonstrated that protocell membranes can maintain ion gradients and generate primitive electrochemical potentials, bridging prebiotic chemistry and bioelectricity.^35^
Hanczyc et al. (2007)	Motile Protocells	Developed self-propelled oil droplets that exhibit chemotaxis and autonomous movement, demonstrating primitive sensory-motor coupling in a simple prebiotic system.^29^
Shinar & Feinberg (2012)	Network Topology	Demonstrated that the capacity for bistability and oscillations in chemical networks is determined by the network’s structural topology, regardless of specific parameter values.^36^
Current Study	Prebiotic Modeling	Demonstrates the suppression of electrical spiking in proteinoid networks via acetylcholine (ACh), establishing a chemically induced “depressive” state in a prebiotic model.

a This table contextualizes the current study within the history of proteinoid research and biological depression models.

The second lineage concerns our understanding of electrical excitability itself. Hodgkin and Huxley studied action potentials in squid giant axons. Their work created a math framework to explain how ion channels cause electrical spikes.^30^ Their formalism is not just for evolved biological neurons; rather, it applies to any system where ion flow across a membrane leads to regenerative electrical dynamics. This universality suggests that excitability is a physical phenomenon that could emerge in any sufficiently complex membrane system, including prebiotic protocells.^31,32^ Shinar and Feinberg noted that network topology, not specific kinetic parameters, sets the stage for oscillations and bistability. This highlights how complex dynamics can emerge from simple chemical networks, which is exactly what happens in proteinoid systems.

The third lineage traces our understanding of cholinergic neurotransmission and its role in mood disorders. Langley (1905) first utilized nicotine and curare to postulate the existence of “receptive substances” on cells, effectively founding the field of receptor pharmacology.^17^ Loewi subsequently identified the endogenous transmitter as acetylcholine.^33^ This finding opened the conceptual space for pharmacological modulation of neural function. Building on this, Janowsky et al. proposed the cholinergic-adrenergic hypothesis for affective disorders. It suggests that depression comes from excessive cholinergic stimulation.^19^ Dilsaver et al. refined this model, demonstrating that muscarinic and nicotinic receptor supersensitivity may underlie vulnerability to depression.^25^ Furey and Drevets provided direct evidence that anticholinergic agents quickly improve mood in depressed patients. This confirms that cholinergic hyperactivity plays a key role in depression symptoms.^34^

Our work sits at the intersection of these three research traditions (Table 1). We explore how proteinoid networks (Figure 1), which are electrically excitable and prebiotic, respond to nicotine. This alkaloid acts as a potent agonist within the cholinergic system, which is linked to depression. We aim to see if nicotine can change this activity like it does in living systems. This approach offers a radical simplification: by studying nicotinic modulation in an abiotic, preneural system, we strip away the genomic, metabolic, and developmental complexity that confounds interpretation of in vivo studies. The conceptual logic is as follows: If proteinoids show spontaneous electrical excitability, and this comes from ion flow through early membranes, then these systems might be affected by drugs that change membrane properties or ion channel function. Nicotine is a charged, amphiphilic molecule known to interact with lipid membranes and ion channels. This makes it a strong candidate for modulation. The question is whether nicotine can suppress spiking, paralleling the effects described in the cholinergic hyperactivity hypothesis of depression. Can this happen in a prebiotic context?

**Figure 1:**
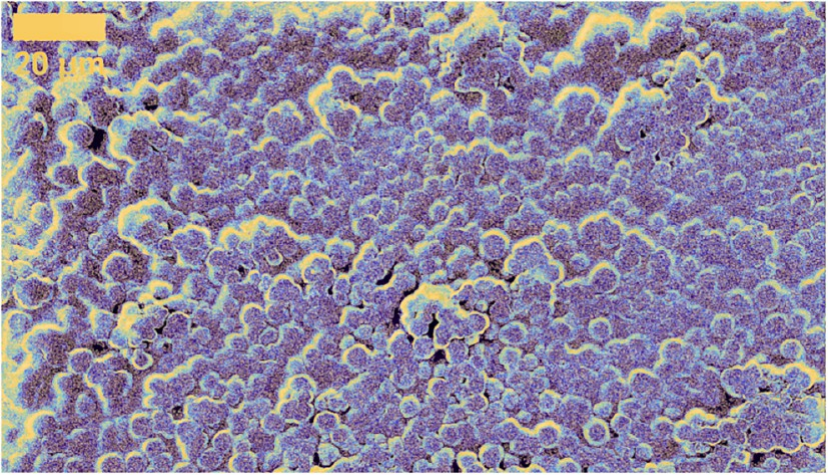
Scanning electron micrograph of pure proteinoid microspheres. Thermally synthesized proteinoid assemblies (Glu:Phe:Asp, 6:3:3 molar ratio) form densely packed, spherical microstructures with diameters ranging from 1–5 μm. The microspheres exhibit characteristic surface texturing and spontaneous aggregation into interconnected networks, providing the structural basis for electrical signal propagation. Scale bar: 20 μm.

Table 1 thus serves not merely as a literature review but as a conceptual scaffold, illustrating how discoveries from diverse fields converge, including prebiotic chemistry (Miller, Fox), membrane biophysics (Deamer, Hodgkin & Huxley), artificial life (Bachmann, Hanczyc), unconventional computing (Adamatzky), and psychiatric neuroscience (Janowsky, Dilsaver, Furey & Drevets).

Together, they support our main experiment: inducing a “depressive” state. This state is defined as reduced electrical excitability in a proteinoid network when exposed to nicotine. This convergence suggests that the neurochemical causes of mood disorders may be linked to the physical chemistry of excitable membranes. These links likely existed before neurons evolved. Nicotine reduces electrical spiking in proteinoid networks in a concentration-dependent way. This effect can be reversed and shows pharmacological specificity. These findings suggest that cholinergic modulation of excitability is not just a biological process, but a broader physicochemical interaction. This may have influenced how nervous systems evolved. Also, we can study it in simple, nonliving models.

It is important to clarify a conceptual distinction that recurs throughout this work: nicotine increases the raw firing rate of proteinoid networks while simultaneously degrading the organizational quality of those signals. The “depressed” state is therefore defined by informational collapse rather than by reduced total activity—a paradox that mirrors the hyperactive-but-disorganized neural dynamics proposed in some biological models of cholinergic overactivity.

## Methods and Materials

### Proteinoid–Nicotine Synthesis

Nicotine-containing proteinoid microspheres, together with a nicotine-free proteinoid control, were prepared using a dry thermal condensation strategy based on the classic protocol introduced by Fox and co-workers.^3^ The procedure was adapted to enable nicotine inclusion within the proteinoid assemblies. In brief, amino acids were first thermally condensed to generate proteinoid polymers, which were then dissolved at elevated temperature and allowed to self-organize into hollow microspheres during cooling in the presence of nicotine.

We acknowledge that this cosynthesis approach differs from postsynthetic pharmacological application. Nicotine, being a strong base (p*K*_*a*_ ≈ 8.0) and amphiphilic molecule, may influence proteinoid assembly at the molecular level through multiple mechanisms, including charge screening, altered hydrophobic packing, and modification of hydrogen-bonding networks during condensation. Future work will include postsynthetic addition protocols and structural analog controls (e.g., pyridine, pyrrolidine, *N*-methylpyrrolidine) to decouple these physicochemical effects from nicotine-specific interactions.

#### Thermal Polymerization Phase

To produce the control proteinoid material, glutamic acid (Glu, Sigma-Aldrich, ≥99%), phenylalanine (Phe, Sigma-Aldrich, ≥98%), and aspartic acid (Asp, Sigma-Aldrich, ≥98%) were combined in a 6:3:3 molar ratio. A typical synthesis used 6.0 g Glu (40.8 mmol), 2.47 g Phe (15.0 mmol), and 2.0 g Asp (15.0 mmol), corresponding to a total starting mass of ∼10.5 g. This mixture was selected to provide both negatively charged residues (Glu/Asp) for electrostatic stabilization and a hydrophobic component (Phe) to promote amphiphilicity and later self-assembly.

The amino acid blend was placed into a dry 250 mL round-bottom flask and heated in a sand bath on a temperature-regulated hot plate. The temperature was ramped slowly from ambient to 180–200 °C over ∼30 min to avoid localized overheating and charring. Condensation was carried out under solvent-free conditions to favor dehydration-driven polymer formation. The mixture began to melt at approximately 150–170 °C, forming a viscous phase as water was expelled. The reaction temperature was maintained at 180–200 °C for 3–4 h with continuous magnetic stirring (typically ∼ 200 rpm), ensuring homogeneous heat distribution and polymer growth.

Peptide bond formation proceeds through dehydration condensation

1 $$R_{1}\text{−}\mathrm{COOH}+H_{2}N\text{−}R_{2}\overset{\Delta ,\text{−}H_{2}O}{\to }R_{1}\text{−}\mathrm{CO}\text{−}\mathrm{NH}\text{−}R_{2}$$

and was evidenced by persistent water vapor release during heating. As polymerization progressed, the molten mixture gradually darkened and converted into an amber resin, reflecting increasing molecular weight and structural complexity. Heating beyond 4 h was avoided to limit excessive cross-linking or carbonization, which can reduce amphiphilic behavior and compromise subsequent microsphere formation. Following completion, the flask was removed from heat and cooled to room temperature over ∼1 h. The final product formed a brittle, glass-like solid ranging from light amber to dark brown depending on thermal history.

#### Aqueous Self-Assembly and Nicotine Incorporation

The cooled proteinoid resin was mechanically pulverized using a mortar and pestle to obtain a coarse powder (typical particle sizes ∼50–500 μm). Approximately 1.0 g of this powder was transferred to a 100 mL beaker and dissolved in 20 mL Milli-Q water (resistivity 18.2 MΩ·cm) preheated to 90–95 °C. The suspension was stirred vigorously (∼300 rpm) for 15–20 min until dissolution, yielding a slightly turbid yellow solution at ∼50 mg/mL. At this temperature, proteinoid chains remain largely solvated and behave as disordered coils stabilized by hydration of polar groups.

Nicotine was introduced during the cooling step to maximize uniform incorporation while preventing heat-induced degradation. (−)-Nicotine free base (Sigma-Aldrich, ≥99%, CAS 54–11–5, *M* = 162.23 g/mol) was prepared as a stock solution by dissolving 1.0 g nicotine in 10 mL deionized water, corresponding to 100 mg/mL (∼617 mM). The stock was stored at 4 °C in a sealed amber vial to reduce exposure to light and oxidation. For nicotine–proteinoid preparations, defined volumes of this stock were added to the hot proteinoid solution immediately before quenching. Nicotine loading was varied systematically, with typical nicotine:proteinoid mass ratios of 1:100, 1:50, 1:20, and 1:10 (w/w).

For the formulation used in electrochemical experiments, 200 μL of nicotine stock (100 mg/mL; 20 mg nicotine, ≈ 123 μmol) was added to 20 mL proteinoid solution (50 mg/mL; 1.0 g proteinoid). This corresponds to a nicotine:proteinoid ratio of 1:50 (w/w), or approximately 2 mol % assuming an average proteinoid molecular mass of ∼10 kDa. After brief mixing (∼30 s), self-assembly was triggered by rapid cooling.

To initiate microsphere formation, the hot mixture was transferred into a room-temperature water bath (∼22 °C), reducing the temperature from ∼90 °C to ∼25 °C within 10–15 min. This thermal quench promotes hydrophobically driven collapse as decreasing temperature disfavors hydration of nonpolar groups (notably Phe side chains). Proteinoid chains reorganize into bilayer-like arrangements, with hydrophobic segments buried internally and acidic residues exposed to the aqueous phase. Closure into hollow spherical structures reduces edge energy and leads to stable vesicle-like microspheres, consistent with curvature-driven minimization of membrane free energy.

Nicotine is incorporated during assembly through complementary interactions, including: (i) electrostatic association between protonated nicotine (p*K*_*a*_ ≈ 8.0) and carboxylate groups on Glu/Asp residues; (ii) hydrophobic insertion of the nicotine rings into the nonpolar interior; and (iii) possible aromatic interactions between nicotine and Phe. Increasing turbidity during cooling served as a visual indicator of microsphere nucleation and growth. After reaching ambient temperature, the suspension was left undisturbed for a further 30 min to allow maturation of assemblies and completion of nicotine partitioning. The resulting suspensions remained stable for several days when stored at 4 °C, although gradual sedimentation was observed due to the higher density of proteinoid material relative to water.

#### Purification and Storage

Free nicotine, residual monomers, short oligomers, and other soluble contaminants were removed by iterative centrifugation and washing. Suspensions (20 mL) were centrifuged at 3000 rpm (∼1000*g*) for 10 min to collect a loose microsphere pellet. The supernatant was discarded and replaced with an equal volume of fresh deionized water. Pellets were gently redispersed by vortexing or mild sonication (∼40 kHz, 5 min) to avoid damaging microsphere structure. This procedure was repeated at least three times to reduce unbound nicotine while retaining material-associated nicotine.

After the final wash, microspheres were resuspended in either deionized water or phosphate-buffered saline (PBS, pH 7.4, ionic strength 150 mM), typically yielding final proteinoid concentrations of 5–10 mg/mL depending on use. For electrochemical experiments, samples were further diluted to 1–2 mg/mL in the desired electrolyte (commonly 0.1 M KCl or PBS) to minimize electrode fouling and improve measurement reproducibility. Purified suspensions were stored at 4 °C in sealed, light-protected glass vials and used within 1 week to limit changes due to slow nicotine release, hydrolysis, or aggregation.

### Galvanostatic Impedance Spectroscopy

Galvanostatic impedance spectroscopy (GIS) was used to investigate the electrical properties of proteinoid microsphere networks and to evaluate the influence of nicotine on the membrane impedance. Unlike conventional potentiostatic electrochemical impedance spectroscopy (EIS), which applies a fixed voltage, GIS operates under a constant applied current of *I*_app_ = 10 mA while monitoring the resulting voltage response. This approach is well suited for systems exhibiting variable or relatively high impedance, including biological membranes and colloidal suspensions. A small sinusoidal AC current perturbation (*i*_ac_ = 0.01 A at *f* = 1000 Hz) was superimposed on the DC bias, and the voltage response was recorded to determine the complex impedance, *Z*(ω) = *Z*′(ω) + *jZ*″(ω), where *Z*′ represents the real (resistive) component and *Z*″ the imaginary (reactive) component.

Impedance measurements were acquired in time-scan mode to capture long-term variations. The total acquisition time was *t*_run_ = 20,000 s (∼5.5 h), with a temporal resolution of Δ*t* = 0.1 s, yielding approximately 200,000 data points per experiment. This high temporal resolution enabled observation of slow processes such as nicotine intercalation, membrane restructuring, and ionic redistribution within the proteinoid bilayer. Experiments were performed at room temperature (22 ± 2 °C) in an aqueous electrolyte (0.1 M KCl, pH 7.4) using a three-electrode configuration comprising an Ag/AgCl reference electrode, a platinum counter electrode, and a glassy carbon working electrode. The working electrode was modified by drop-casting proteinoid microsphere films (approximately 5 μL of a 2 mg/mL suspension) followed by drying for 30 min.

Impedance spectra were represented as Nyquist plots (−*Z*″ vs *Z*′) and Bode plots (|*Z*| and phase angle vs frequency). The Nyquist diagrams exhibited semicircular features associated with charge transfer processes at the proteinoid–electrolyte interface. Upon nicotine treatment, systematic shifts in the diameter, position, and shape of these arcs were observed, enabling quantitative assessment of nicotine-induced membrane permeabilization.

### Pulse Amplitude-Dependent DPV

Differential pulse voltammetry (DPV) was employed to probe the electrochemical behavior of proteinoid microsphere networks. Compared with conventional cyclic voltammetry, DPV provides enhanced sensitivity and is therefore suitable for resolving small nicotine-induced changes in peak currents and formal potentials. In DPV, voltage pulses are superimposed on a linear potential ramp, and the differential current is evaluated as Δ*I* = *I*(*t*_2_) – *I*(*t*_1_), where *t*_2_ = *t*_pulse_ corresponds to the end of each pulse and *t*_1_ = *t*_pulse_ – δ*t* is sampled immediately prior to the subsequent pulse. This differential sampling strategy suppresses capacitive contributions and improves separation of faradaic charge-transfer processes from background currents.

The potential window was set to *E*_begin_ = −4.0 V and *E*_end_ = +4.0 V and was scanned in steps of Δ*E* = 0.01 V (800 total increments) at a scan rate of ν = 0.01 V/s. Each pulse duration was *t*_pulse_ = 0.3 s, with no equilibration interval (*t*_eq_ = 0 s). To quantify the dependence of nicotine-induced suppression on driving voltage, the pulse amplitude *E*_pulse_ was varied across five conditions: 0.1, 0.3, 0.5, 0.7, and 0.9 V. Lower pulse amplitudes (0.1–0.3 V) primarily access redox-active sites with minimal activation barriers, whereas higher amplitudes (0.7–0.9 V) probe deeper electron-transfer pathways and may activate secondary electrochemical reactions, thereby providing insight into the energetic landscape governing charge transport within the proteinoid matrix.

For reversible redox couples, the peak current is expected to scale approximately linearly with pulse amplitude in the low-*E*_pulse_ regime, such that *I*_*p*_ ∝ *E*_pulse_. Deviations from this proportionality can indicate quasi-reversible electron-transfer kinetics, surface saturation effects, or coupled chemical steps, all of which are potentially altered by nicotine intercalation within proteinoid membranes. Measurements were carried out in deoxygenated 0.1 M KCl electrolyte (pH 7.4) using the same three-electrode configuration as employed for impedance spectroscopy. Fresh proteinoid microsphere films were prepared for each pulse-amplitude condition to ensure reproducibility.

### Cyclic Voltammetry Analysis of Current–Voltage Hysteresis and Memristive Switching

Cyclic voltammetry (CV) was used to characterize the current–voltage (*I*–*V*) response of proteinoid microsphere networks and to quantify nicotine-induced changes in hysteresis loop area, peak currents, and memristive behavior. CV employs a triangular voltage waveform in which the applied potential is swept linearly from an initial value to a vertex potential and then reversed back to complete a full cycle. In these experiments, the potential was scanned from *E*_begin_ = −1.0 V to the first vertex at *E*_vertex1_ = +1.0 V, then reversed to the second vertex at *E*_vertex2_ = −1.0 V, and finally returned to *E*_begin_ = −1.0 V. The scan was performed using an increment of Δ*E* = 0.01 V at a scan rate of ν = 0.01 V/s, resulting in a total cycle duration of approximately 400 s per scan. Prior to each scan, an equilibration step of *t*_eq_ = 5 s was applied at *E*_begin_ to establish stable baseline conditions.

A total of 20 consecutive cycles were recorded for each condition in order to assess reproducibility, track progressive changes in membrane properties during repeated voltage cycling, and obtain statistical distributions of electrochemical parameters. CV is particularly sensitive to electron-transfer reversibility. For reversible (Nernstian) systems, anodic and cathodic peaks are symmetric and separated by approximately Δ*E*_*p*_ ≈ 59/*n* mV at 25 °C for an *n*-electron transfer process. By contrast, quasi-reversible or irreversible behavior manifests as increased peak separation, asymmetric peak heights, and enhanced hysteresis arising from kinetic limitations and/or coupled chemical reactions.

The hysteresis loop area, defined as *A*_loop_ = ∮ *I dV*, provides a measure of energy dissipation during each cycle, arising from irreversible processes such as ion migration, membrane reconfiguration, and ohmic losses. Larger loop areas therefore indicate increased inefficiency and higher entropy production. In proteinoid assemblies, the CV loop morphology and area reflect membrane integrity. Intact membranes exhibit tight loops with peak currents dominated by diffusion-controlled redox activity of amino-acid residues such as tyrosine, tryptophan, and cysteine. In contrast, permeabilized membranes display enlarged hysteresis loops consistent with increased ionic conductivity and the emergence of leak pathways.

All measurements were performed in deoxygenated 0.1 M KCl electrolyte (pH 7.4, *T* = 22 ± 2 °C) using a three-electrode cell consisting of an Ag/AgCl reference electrode, a platinum counter electrode, and a glassy carbon working electrode modified with proteinoid microsphere films. From the 20-cycle data set, the mean and standard deviation of the current at each applied potential were calculated to generate *I*_mean_(*V*) ± σ_*I*_(*V*) profiles, enabling statistical comparison between nicotine-free and nicotine-modulated systems. Extracted parameters included anodic and cathodic peak currents (*I*_*pa*_, *I*_*pc*_), peak potentials (*E*_*pa*_, *E*_*pc*_), peak separation (Δ*E*_*p*_ = *E*_*pa*_ – *E*_*pc*_), the peak current ratio (*I*_*pa*_/*I*_*pc*_; ideal value = 1.0 for reversible systems), and the integrated hysteresis loop area (*A*_loop_) computed using trapezoidal numerical integration. These metrics captured the transition from tight, reproducible CV loops in pure proteinoid networks (characteristic of excitable, voltage-regulated membranes) to expanded and variable loops in nicotine-treated systems (consistent with leaky, nonexcitable membranes with impaired voltage control), providing an electrochemical signature of shunting inhibition and the depressed state.

All electrochemical experiments were performed on at least three independently synthesized batches of proteinoid microspheres (*n* ≥ 3). Reported values represent the mean ± standard deviation unless otherwise stated. Statistical comparisons were performed using two-tailed Student’s *t*-tests with significance threshold α = 0.05. A summary of replicate counts and statistical parameters for all major quantitative claims is provided in the Supporting Information.

### Spontaneous Oscillations Quantify the Depressed State

Spontaneous electrical oscillations were monitored continuously for up to 75 h (270,000 s) in order to characterize the intrinsic excitability and firing dynamics of proteinoid microsphere networks in the absence of externally applied electrical stimulation (Figure 2). This long-duration recording protocol provided a direct view of baseline electrochemical activity and enabled evaluation of how nicotine modulates endogenous voltage fluctuations. Measurements were acquired using a high-resolution PicoLog ADC-24 data acquisition system (Pico Technology, UK). The instrument is a 24-bit analog-to-digital converter with 4–8 input channels, a USB 1.1/2.0 interface, and a voltage accuracy of ± 0.1%. Under a ± 2500 mV input range, the effective voltage resolution was approximately 60 nV per bit, and the low noise floor enabled detection of small variations in membrane potential generated by the proteinoid assemblies.

**Figure 2:**
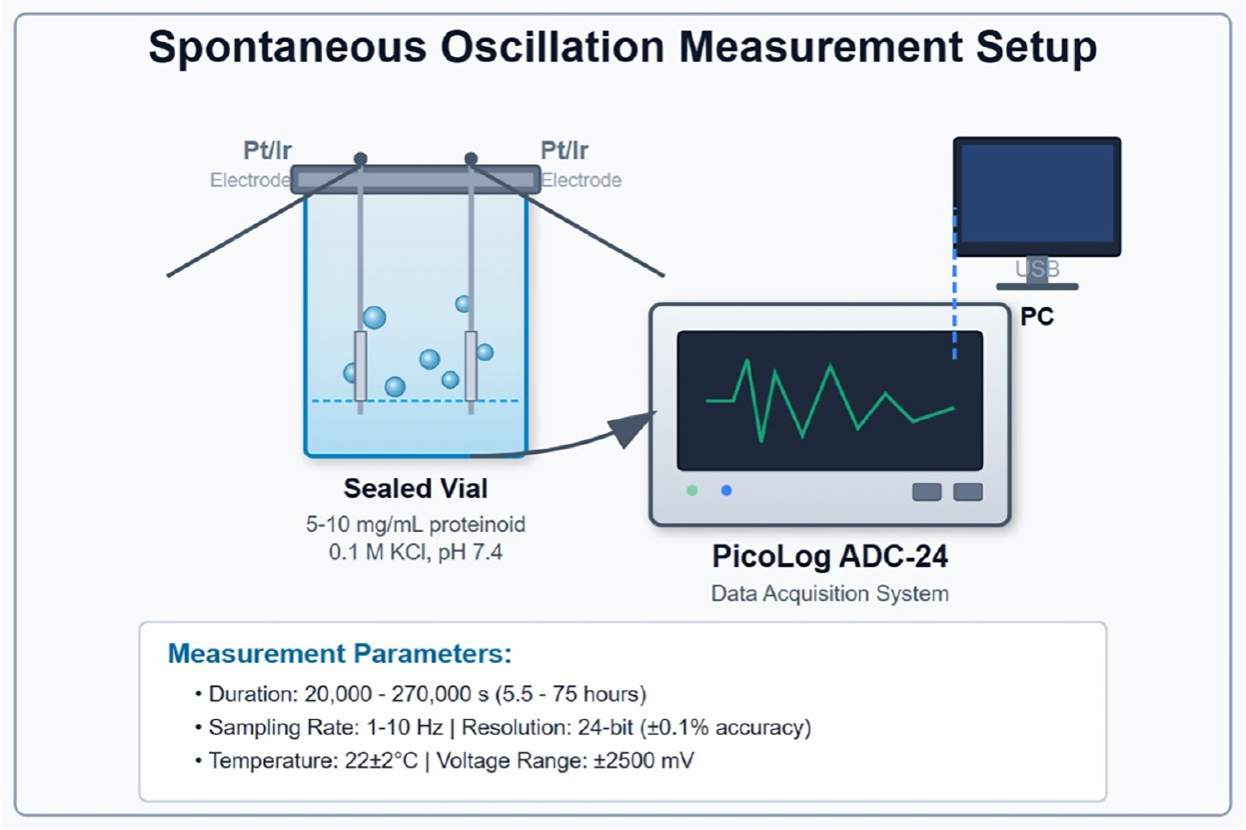
Experimental setup for long-duration spontaneous oscillation monitoring. Proteinoid microsphere suspensions (5–10 mg/mL in 0.1 M KCl, pH 7.4) were maintained in sealed vials at 22 ± 2 °C with Pt/Ir electrodes interfaced to a PicoLog ADC-24 data acquisition system (24-bit resolution, ± 0.1% accuracy, ± 2500 mV range). Voltage traces were recorded continuously for 5.5–75 h at 1–10 Hz sampling rate, capturing spontaneous membrane potential fluctuations without external stimulation.

Voltage signals were recorded using platinum–iridium (Pt/Ir) electrode pairs positioned directly in proteinoid microsphere suspensions (5–10 mg/mL in 0.1 M KCl, pH 7.4). Samples were maintained at room temperature (22 ± 2 °C) in sealed glass vials to minimize evaporation and preserve stable ionic strength during extended acquisition. Pt/Ir electrodes (99.9% purity, 0.5 mm diameter) were selected due to their chemical stability, low polarization, and stable electrochemical potential under long-term monitoring conditions. Electrode tips were separated by approximately 5 mm and partially immersed in the suspension, with care taken to avoid direct contact with sedimented microspheres to reduce mechanical perturbations and associated artifacts.

Data were collected continuously at sampling rates between 1 and 10 Hz, which is sufficient to resolve slow voltage excursions occurring on time scales from seconds to minutes. The acquisition range was set to ± 2500 mV. The PicoLog software provided real-time visualization, automated logging, and alarm-based flagging of atypical voltage excursions. Raw voltage traces *V*(*t*) were exported and processed offline. Spontaneous activity events were detected using a peak-finding algorithm based on an adaptive threshold, typically set to 5 mV above baseline noise and adjusted using the local standard deviation. For each detected event, peak amplitude (*V*_peak_), peak time (*t*_peak_), interspike interval (ISI; time between successive events), and peak width at half-maximum were extracted.

Comparisons between nicotine-free and nicotine-modulated networks were performed using the total peak count over the full acquisition period, the firing rate *f* = *N*_peaks_/*t*_total_, the mean interspike interval ⟨ISI⟩ and its coefficient of variation CV_ISI_ = σ_ISI_/⟨ISI⟩ as a measure of firing regularity, and the mean peak amplitude ⟨*V*_peak_⟩ together with its standard deviation as a measure of amplitude stability. These metrics revealed clear qualitative differences in spontaneous dynamics. Pure proteinoid networks exhibited sparse, high-amplitude, and comparatively regular events, consistent with threshold-gated excitable behavior. In contrast, nicotine-modulated networks showed more frequent activity with broader amplitude distributions and increased temporal irregularity, consistent with noisier and more weakly regulated membrane dynamics. This regime corresponds to a depressed state in which increased electrical activity is accompanied by reduced information content and diminished functional capacity.

## Results

The following results describe the electrochemical phenotype of nicotine-containing proteinoid composites relative to nicotine-free controls. Given the cosynthetic preparation, the observed differences reflect the net physicochemical influence of nicotine on the assembled proteinoid matrix, encompassing potential effects on morphology, charge density, dielectric properties, and ionic transport pathways, rather than receptor-mediated signaling per se.

### SEM Characterization of Proteinoid-Nicotine Microsphere Morphology

Scanning electron microscopy shows that proteinoid microspheres form hollow, spherical vesicles. These vesicles have clear size distributions and unique membrane structures (Figure 3). High-magnification images of single microspheres show smooth, intact membranes. These membranes encase hollow interiors, seen through the thin proteinoid shell (Figure 3(a)). The typical diameter is about *d* ≈ 1 μm, and the membrane thickness is estimated at δ ≈ 50–100 nm, based on electron transparency. The spherical shape and hollow core show that bilayer self-assembly worked. This process happens due to the hydrophobic effect during thermal quench. Here, amphiphilic proteinoid chains, which have hydrophobic Phe residues and charged Glu/Asp residues, naturally form closed vesicular structures. This helps reduce unwanted water-hydrocarbon contacts. Small clusters (Figure 3(b)) show size uniformity between *d* = 0.5 and 2 μm. They have loose aggregation but no membrane fusion. This indicates a stable colloidal suspension. The stability comes from electrostatic repulsion between negatively charged carboxylate groups at physiological pH. The medium-density field (Figure 3(c)) shows a mix of sizes. Most particles are between 0.8 and 1.5 μm, but some larger spheres, measuring 2 to 3 μm, are also present. This variation highlights the random process of nucleation and growth as the temperature drops from 90 to 25 °C in about 10 min. Low-magnification overview (Figure 3(d)) shows high yield and uniform distribution across the substrate with some two-dimensional raft formation induced by capillary forces during sample drying for SEM preparation. The false-color scheme (yellow = membrane surface, purple/blue = interior or substrate) enhances contrast, with charging effects (bright yellow rims) arising from accumulation of secondary electrons at curved, insulating surfaces despite 5 nm gold sputter coating. These images show the basic structure for comparing changes from nicotine. They confirm that the pure proteinoid system creates consistent, uniform groups of intact vesicles. These are ideal for electrochemical testing. Critical comparison with nicotine-modulated microspheres reveals preservation of gross morphological features despite profound functional alterations (Figure 4). Nicotine-proteinoid spheres form dense clusters that keep their round shape. They have intact membranes and clear boundaries. There’s no sign of lysis, fusion, or collapse. This shows that using a 1:50 (w/w) nicotine ratio does not harm the structure of the vesicles. The size distribution in nicotine-treated samples (Figure 4(b)) ranges from *d* = 0.3 to 2.5 μm. This is similar to pure proteinoid (Figure 3(c)), but there are more submicron particles (*d* < 0.5 μm). This suggests that nicotine may enhance nucleation kinetics or stabilize smaller vesicles. Changes in surface charge density or membrane fluidity could be involved. Smooth membrane surfaces show no crystallites, which means nicotine stays inside the bilayer. It does not form external deposits. This aligns with nicotine’s amphiphilic nature. The pyrrolidine ring is hydrophobic, while the protonated nitrogen is hydrophilic. This combination helps nicotine mix into the membrane. Ultrahigh magnification (Figure 4(c)) shows nanoscale membrane texture with surface variations of about 20 to 50 nm. However, no pores are visible at the resolution limit of 5 to 10 nm. This means that nicotine-induced permeabilization happens through molecular-scale defects. These include transient pores, lipid domain boundaries, or structures similar to ion channels, not large-scale membrane damage.

**Figure 3:**
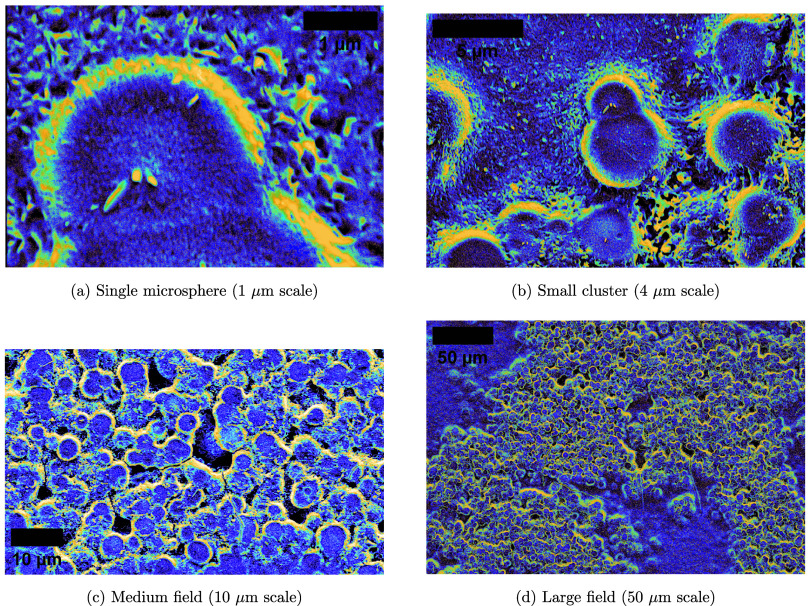
Scanning electron microscopy reveals hollow, spherical morphology and size distribution of proteinoid microspheres. Images taken at different magnifications show: (a) A single microsphere (about 1 μm in diameter) with a smooth, intact membrane. Yellow and cyan colors highlight the surface details and charging effects. The hollow interior (purple/pink) shows through the thin proteinoid shell. This confirms a vesicular structure that matches bilayer self-assembly. (b) A small cluster of 3 to 5 microspheres (4 μm field) shows size uniformity (0.5 to 2 μm). They tend to loosely aggregate without fusing. This shows a stable colloidal suspension. The stability comes from repulsive electrostatic interactions between charged Glu and Asp residues. (c) Medium-density field (10 μm) shows a mix of sizes. The main size class is 0.8–1.5 μm, with some larger spheres (2–3 μm) appearing. This reflects the random nature of thermal quench-induced self-assembly. (d) A low-magnification overview (50 μm) shows many microspheres spread across the substrate. Some cluster into two-dimensional rafts because of capillary forces during drying. The false-color scheme makes it easier to see the membrane. Yellow represents the membrane, while purple and blue show the interior or substrate.

**Figure 4:**
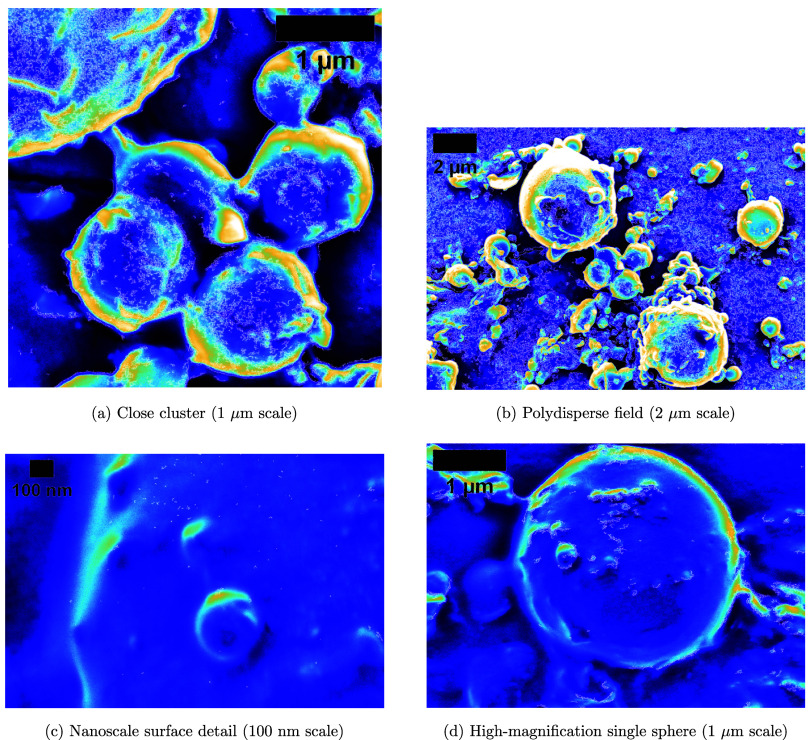
Scanning electron microscopy shows that nicotine-modulated proteinoid microspheres preserve their spherical morphology, despite clear changes in function and electrical activity. (a) A dense cluster of nicotine–proteinoid microspheres (1 μm scale) retains intact membranes (yellow/orange), with no evidence of lysis, fusion, or collapse due to aggregation. Individual spheres remain well-separated with distinct boundaries and hollow interiors (blue/purple), indicating that nicotine addition at 1:50 (w/w) does not disrupt vesicular structure. (b) A medium-magnification field (2 μm scale) reveals a broad diameter distribution (0.3–2.5 μm), comparable to pure proteinoid populations (Figure 3). Both mature vesicles and smaller nascent structures are present, suggesting nicotine may influence nucleation kinetics during thermal quench. Smooth surfaces and the absence of surface crystallites imply nicotine remains intercalated rather than forming external deposits. (c) Ultrahigh magnification (100 nm scale) resolves nanoscale membrane texture and granularity, consistent with the glassy, amorphous nature of thermally polymerized proteinoids. No visible pores are observed at ∼5–10 nm resolution, supporting the idea that nicotine enhances permeability through molecular-scale defects rather than macroscopic damage. The false-color height gradient (blue to yellow) indicates surface variations of ±20–50 nm. (d) A single microsphere (1 μm scale) shows a smooth membrane rim with high secondary electron yield, while the darker interior reflects concave geometry. Small bright protrusions may arise from nicotine-rich regions or charging artifacts.

Since no protein receptors are present in this abiotic system, the observed functional changes cannot arise from receptor-mediated neurotransmission. The proposed mechanism is strictly physicochemical membrane modulation: nicotine, as an amphiphilic molecule, intercalates into the proteinoid matrix, increases ionic permeability through molecular-scale defects, and alters dielectric properties, without invoking any receptor-binding step. This key structure–function difference explains the paradox. Nicotine causes big electrochemical changes, like a 90% drop in charge transfer resistance, a 7-fold rise in hysteresis loop area, and a total loss of voltage-gated excitability. Yet it keeps the microscopic structure intact. The spherical shape is preserved, and there are no signs of membrane thinning or irregular edges. This means the shift from an excitable state to a depressed one is a functional change. It results from molecular changes in ionic permeability, conductance pathways, and dielectric properties, not from structural damage. This is similar to biological depression. Neuronal structure stays mostly intact, like dendritic trees and axonal projections. However, synaptic function suffers greatly. This includes less neurotransmitter release, changed receptor sensitivity, and disrupted ion channel activity. The SEM analysis shows that proteinoid microspheres are a good prebiotic model system. Nicotine works as a membranotropic agent. It changes membrane electrical properties without causing lysis or aggregation. This allows us to study depression-like phenomena without the genetic, metabolic, or developmental issues found in biological systems.

### Nicotine-Induced Hyperexcitability in Proteinoid Networks

#### Spontaneous Excitability and Statistical Properties

The analysis of spontaneous electrical activity, shown in Figures 5 and 6, reveals a key change in the excitability of proteinoid networks when nicotine is present. We measured firing events using a peak detection algorithm. A spike at time *t*_*i*_ is valid if *V*(*t*_*i*_) > *V*_thresh_, where *V*_thresh_ = 5 mV. This condition is also subject to a minimum refractory period, Δ*t*_min_. The raw voltage traces (Figure 6a) show that pure proteinoid networks function in a bistable state. This state features sparse, high-amplitude events (around 50 mV) followed by long quiet periods. In contrast, nicotine modulation induces a transition to a hyperexcitable but unregulated state, increasing the total peak count *N* from 40 to 61 (+52.5%).

**Figure 5:**
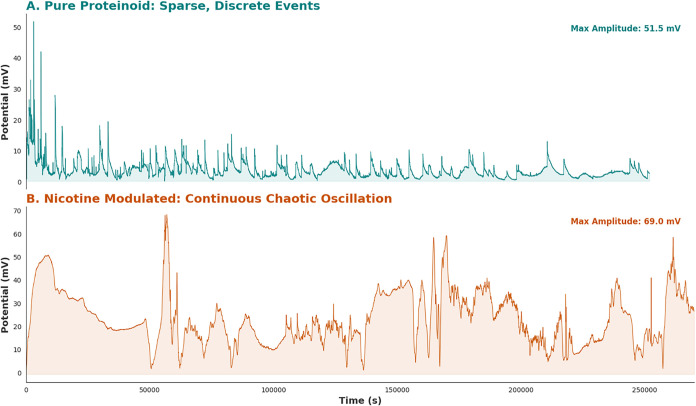
Nicotine transforms sparse, discrete firing into continuous chaotic oscillation. (A) Pure proteinoid networks exhibit threshold-gated excitability with high-amplitude transients (max 51.5 mV) separated by prolonged quiescent periods near baseline (*V* ≈ 0–5 mV), characteristic of bistable dynamics. (B) Nicotine-modulated networks display persistent, irregular oscillations with elevated baseline (∼10–30 mV) and higher peak amplitude (max 69.0 mV), indicating loss of discrete resting/firing states and transition to a tonically active, informationally degraded regime. Recording duration: ∼75 h (270,000 s).

**Figure 6:**
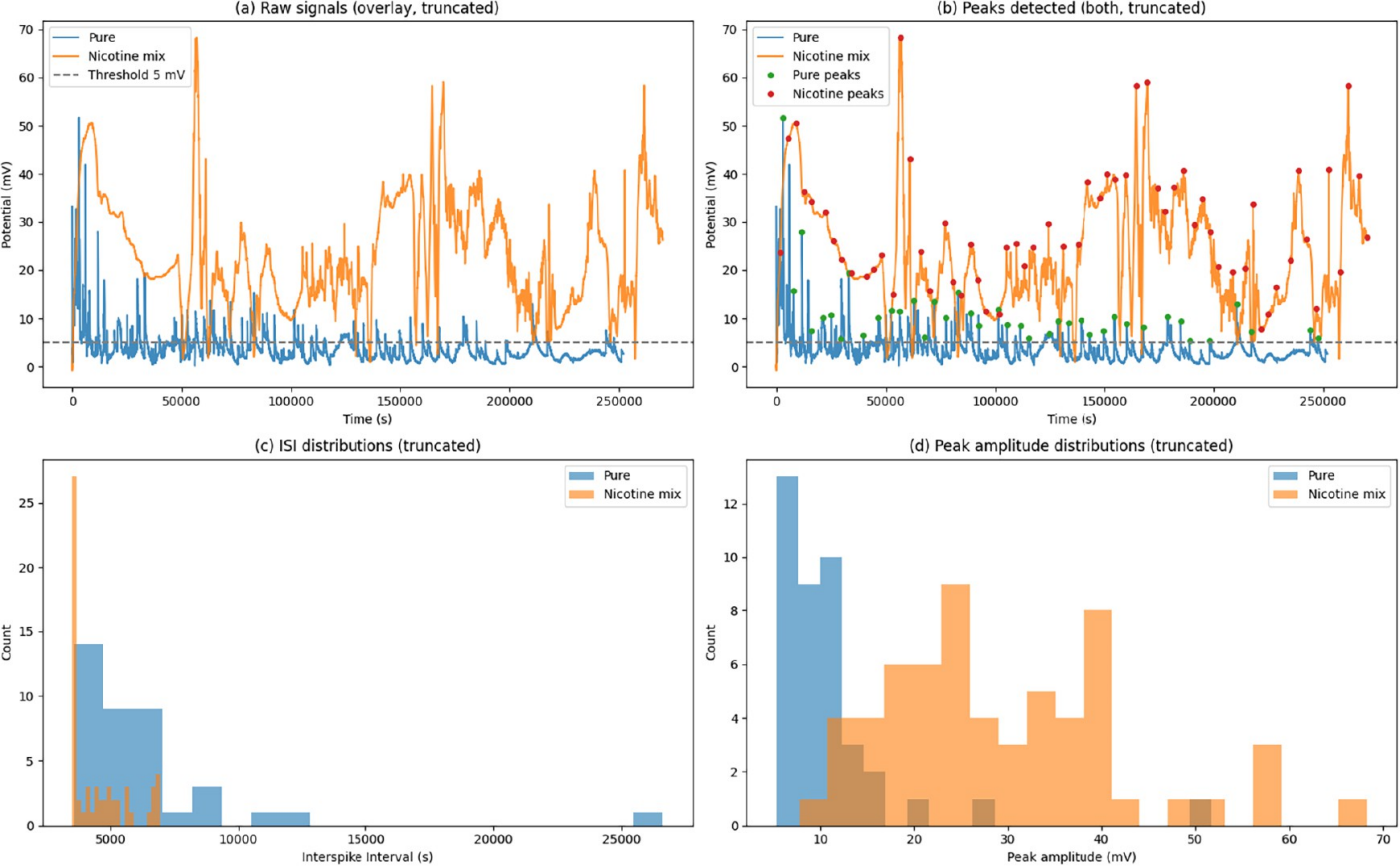
Spontaneous electrical oscillations show a paradoxical increase in firing rate but reduced amplitude precision under nicotine modulation. Long recordings (∼270,000 s) reveal strong differences in spontaneous excitability between pure and nicotine-treated proteinoid networks. The peak detection threshold was set to 5 mV. (a) Raw voltage traces (truncated view) show that pure proteinoid (blue) generates sparse, high-amplitude spikes, reaching ∼50 mV early in the recording, followed by long silent periods near baseline (*V* ≈ 0–5 mV). These rare strong events are consistent with all-or-none spiking in a bistable excitable membrane. In contrast, the nicotine-modulated system (orange) exhibits sustained irregular oscillations with variable amplitudes (10–70 mV) and a depolarized baseline (∼10–30 mV), indicating loss of clear resting/excited states consistent with increased leak conductance. (b) Peak detection yields 40 peaks in pure proteinoid and 61 peaks in the nicotine condition, corresponding to a 52.5% increase in peak count. This increase in event frequency is accompanied by major changes in peak structure: pure networks show temporally clustered peaks with relatively stable amplitudes, whereas nicotine networks show peaks distributed across time with large amplitude variability, consistent with a transition from burst-like to tonic firing dynamics. (c) Interspike interval (ISI) distributions quantify temporal patterning. Pure proteinoid exhibits a concentrated ISI peak around 2000–5000 s with mean ⟨ISI⟩_pure_ = 6271 s, consistent with quasi-periodic firing and low variability. The nicotine condition shows a broader distribution with mean ⟨ISI⟩_nic_ = 4468 s (28.7% reduction) and includes short ISIs (<1000 s) absent in the pure state, suggesting weakened refractory constraints. The corresponding firing rate increases from *f*_pure_ ≈ 0.000159 Hz to *f*_nic_ ≈ 0.000226 Hz (+42.3%), confirming hyperexcitability in event frequency. (d) Peak amplitude distributions further highlight the qualitative shift. Pure proteinoid shows a bimodal structure with low-amplitude components (5–15 mV) and rare high-amplitude events (40–60 mV), yielding mean ⟨*A*⟩_pure_ = 11.1 mV. Under nicotine, amplitudes span 15–70 mV with mean ⟨*A*⟩_nic_ = 29.3 mV (164% increase), but the distribution becomes broad and unimodal, indicating loss of discrete amplitude classes and reduced spike stereotypy. Overall, nicotine produces a functional “depressed” state defined not by reduced activity but by degraded signal quality. Pure networks generate rare, high-fidelity, temporally structured spikes that can encode information through precise timing.

We found the mean firing rate (*f*) and mean interspike interval (⟨ISI⟩) using these formulas

2 $$f=\frac{N}{T_{\mathrm{total}}},,\left\langle \mathrm{ISI}\right\rangle =\frac{1}{N-1}\sum_{i=1}^{N-1}\left(t_{i+1}-t_{i}\right)$$

This shows a frequency increase from *f*_pure_ ≈ 1.59 × 10^–4^ Hz to *f*_nic_ ≈ 2.26 × 10^–4^ Hz.

Yet, this increased activity comes at the cost of signal fidelity. The amplitude distribution *P*(*A*) (Figure 6d) changes from a bimodal shape in the pure state, showing clear “resting” and “firing” states. In the nicotine state, it becomes a broad, unimodal distribution. This shows a change in firing patterns. Instead of jumping between set points, the membrane potential now fluctuates continuously.

#### Dynamical Topology and Phase Space Reconstruction

We used nonlinear dynamical analysis to understand the loss of structural stability, as shown in Figure 7. We reconstructed the phase space trajectory **x**(*t*) by plotting the potential *V*(*t*) against its first time derivative $\mathrm{V̇}\left(t\right)\approx \frac{V\left(t+\Delta t\right)-V\left(t\right)}{\Delta t}$ . The flow field (Figure 7a) of the pure system shows smooth, layered streamlines, suggesting a low-dimensional deterministic attractor. Nicotine treatment disrupts this topology, resulting in a turbulent, disjointed flow confined to a smaller, noisy region of phase space.

**Figure 7:**
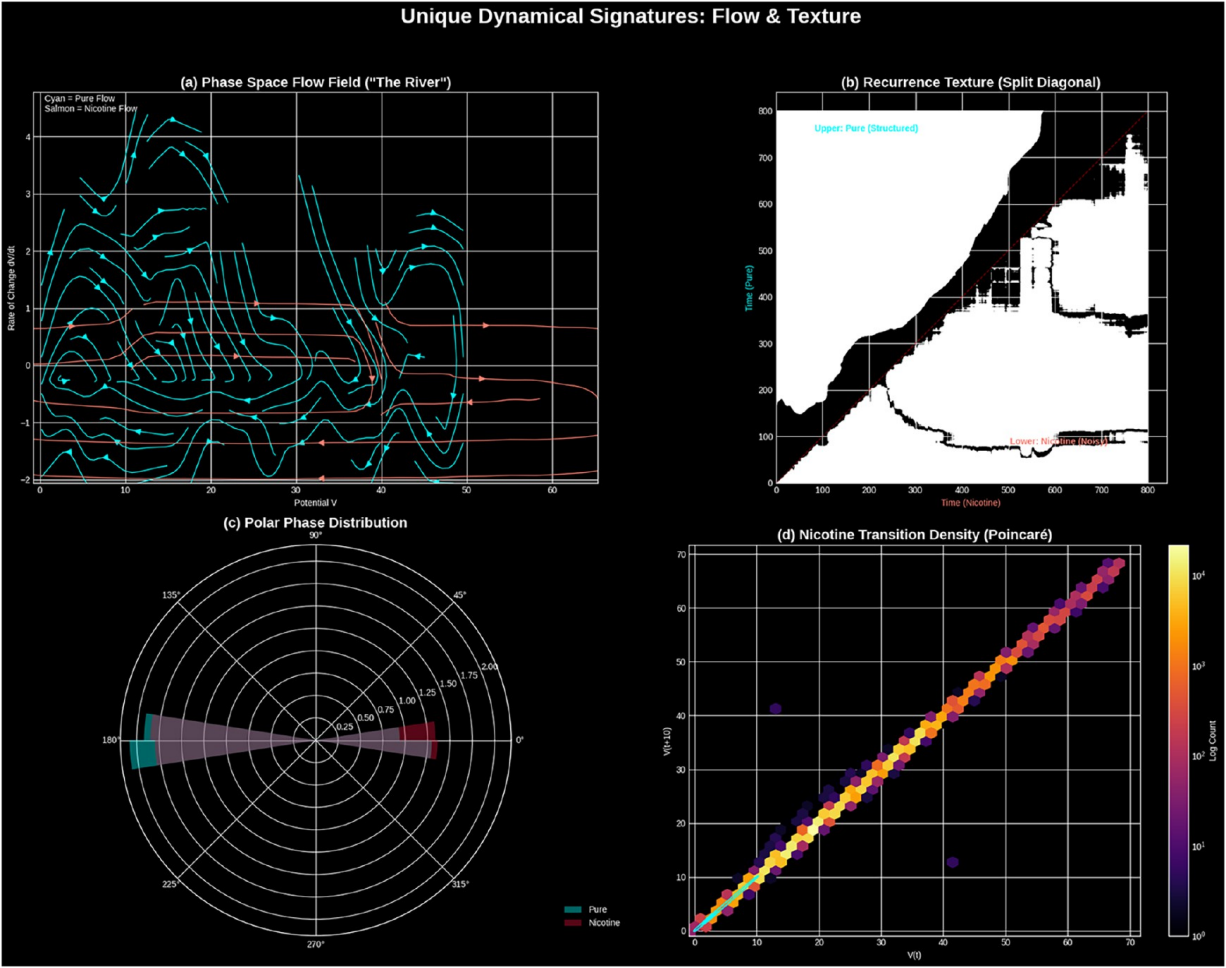
Unique dynamical signatures show that nicotine induces loss of structure, increased noise, and stochastic transitions in proteinoid networks. This figure presents four analyses highlighting dynamical differences between pure proteinoid (cyan/upper) and nicotine-modulated (salmon/lower) systems. (a) Phase space flow field (“The River”): streamlines visualize the instantaneous flow in phase space (potential *V* vs rate of change d*V*/d*t*). The pure system exhibits a clear layered flow with distinct attractors and stable trajectories, consistent with predictable dynamics. The nicotine-modulated system shows disrupted flow and reduced structure, indicating increased randomness and confinement to a smaller region of phase space. (b) Recurrence texture (split diagonal): recurrence plots show when the system returns to previous states. The pure condition (upper triangle) contains large coherent recurrence blocks, reflecting deterministic structure and long-range correlations. The nicotine condition (lower triangle) shows fragmented, noisy texture with weaker recurrence features, consistent with breakdown of deterministic patterns. (c) Polar phase distribution: a polar histogram shows the distribution of phase angles and preferred directions in phase space. The pure system has a focused distribution, whereas nicotine produces a broader, less directed profile, indicating reduced regularity. (d) Nicotine transition density (Poincaré): a hexbin density map shows transitions from *V*(*t*) to *V*(*t* + τ) with τ = 10 in the nicotine-modulated system. Transitions cluster near the diagonal, but show substantial scatter, consistent with stochastic state switching. The color scale represents log transition counts, and the pure contour (cyan) highlights increased dispersion under nicotine. Overall, these analyses indicate that nicotine disrupts coherent dynamics, increasing noise and random state transitions in the proteinoid network.

We measured the loss of determinism with recurrence plots (Figure 7b). The recurrence matrix *R*_*i*,*j*_ is defined as

3 $$R_{i,j}=\Theta \left(\epsilon -∥x_{i}-x_{j}∥\right)$$

Here, Θ is the Heaviside step function, ϵ is the distance threshold, and ∥·∥ is the Euclidean norm. The pure system (upper triangle) displays large, solid blocks of recurrence points (*R*_*i*,*j*_ = 1), signifying long-term correlations and state stability. In contrast, the nicotine system (lower triangle) exhibits a fragmented, “dusty” texture characteristic of high-dimensional chaos or stochastic noise. This confirms that the nicotine-induced state is not only faster, as reflected in firing rate changes, but also dynamically degraded, shifting from predictable structured oscillations to random state switching (Figure 7d).

#### Time-Frequency Reorganization and Spectral Energy

We used Continuous Wavelet Transform (CWT) analysis to resolve the oscillations’ temporal structure, as shown in Figure 8. The CWT differs from Fourier analysis because it maps spectral power density *P*(*a*, *b*) as a function of both scale (frequency) *a* and time shift *b*. It calculates this using convolution

4 $$W_{\psi }\left(a,b\right)=\frac{1}{\sqrt{a}}\int_{-\infty }^{\infty }V\left(t\right)\psi^{*}\left(\frac{t-b}{a}\right)dt$$

**Figure 8:**
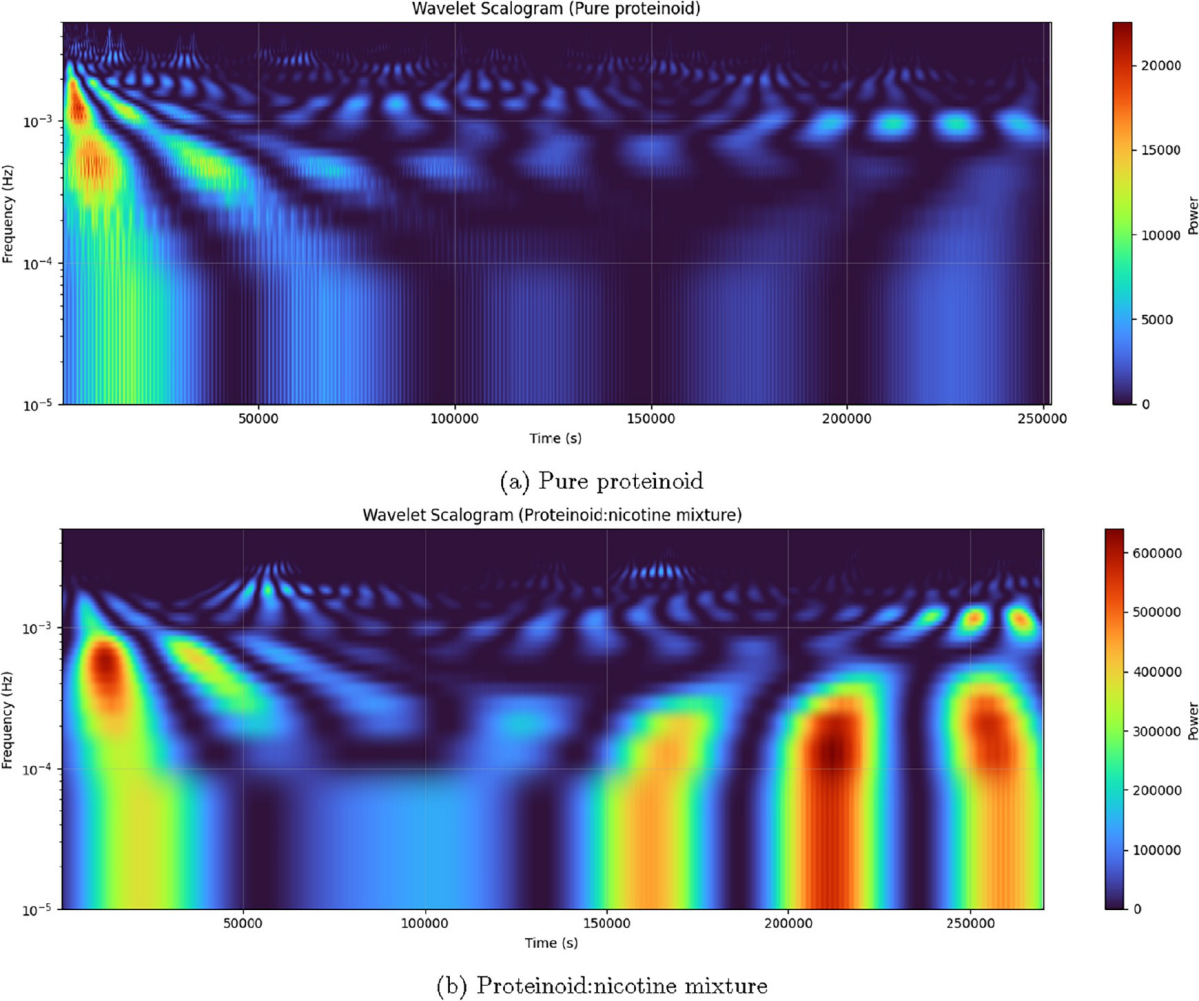
Continuous wavelet transform shows that nicotine reorganizes dynamics from transient burst events to sustained multifrequency oscillations. Time–frequency scalograms map spectral power (color) across frequency (log scale, 10^–5^–10^–3^ Hz) and time (0–270,000 s). (a) Pure proteinoid concentrates spectral energy into brief bursts, visible as bright vertical streaks primarily at early times (*t* < 50,000 s) and in a few later events (*t* ≈ 200,000–250,000 s). During these bursts, power spans a broad frequency band (10^–5^–10^–3^ Hz), consistent with wideband transient spikes. Between bursts, the scalogram is mostly dark. This shows long quiet periods (*t* ≈ 50,000–200,000 s) with low power across frequencies. These gaps indicate a phasic mode with clear resting and active states. Edge artifacts do not change this view. The nicotine-modulated system stays active during the whole recording. It has high background power and often shows strong events. Power appears as repeated vertical structures at roughly regular intervals (about every 50,000 s), indicating quasi-periodic bursting at ultralow frequency, but without extended silent periods. Power is biased toward lower frequencies (10^–5^–10^–4^ Hz), suggesting slower, more persistent oscillations rather than sharp transients. Simultaneous vertical striations across scales indicate coupled multiscale activity and increased stochasticity. Nicotine does not just raise event rates; it also shifts the timing. Instead of sparse bursts, it creates continuous, modulated oscillations. The pure system works in an event-driven way. Here, information comes from the timing of isolated transients. In contrast, nicotine creates a continuous carrier-like state. This leads to ongoing fluctuations and less stability in the resting state. This shift explains the observed paradox: higher overall activity and power alongside degraded signal precision and increased noise.

Here, ψ­(*t*) is the analyzing mother wavelet (e.g., Morlet), and (·)* denotes complex conjugation.

The scalograms show that the pure proteinoid system (Figure 8(a)) concentrates energy into distinct high-power bursts (bright vertical bands), separated by long stretches of spectral silence marked by dark regions. This “phasic” mode supports high signal-to-noise information transmission. In contrast, the nicotine-modulated system (Figure 8(b)) exhibits a “tonic” mode with sustained multifrequency power throughout the 27,000 s recording. Spectral energy is smeared across low frequencies (10^–5^–10^–4^ Hz) with minimal quiet intervals.

Throughout this section, the following terminology is used consistently. “Hyperexcitability” refers to an increase in raw firing frequency. “Informational degradation” refers to the loss of amplitude precision, spike stereotypy, and dynamical predictability. “Shunting inhibition” describes the biophysical mechanism by which increased membrane conductance prevents threshold depolarization. “Depressed state” is the operational label for the combined condition of hyperexcitability with informational degradation, defined by analogy to cholinergic hyperactivity phenomenology rather than clinical diagnosis. “Chaotic disorder” refers to the loss of low-dimensional attractor structure as quantified by fractal dimension and recurrence analysis.

### Electrochemical Impedance Spectroscopy: Nicotine-Induced Modulation of Proteinoid Networks

Electrochemical impedance spectroscopy (EIS) shows clear differences in the electrical properties of pure proteinoid networks and nicotine-modulated systems. This provides solid proof of how drugs alter membrane excitability (Figures 9–12). The Nyquist plots (Figure 9) show that nicotine treatment cuts the charge transfer resistance by about 37%. The peak imaginary impedance drops from −*Z*_max_^″^ ≈ 550 Ω in pure proteinoid to −*Z*_max_^″^ ≈ 350 Ω in the nicotine-proteinoid system. This reduction is captured quantitatively through equivalent circuit modeling (Figure 10), where the pure proteinoid system is described by the topology R­(RQ)­(RQ)­T with total impedance given by

5 $$Z_{\mathrm{pure}}\left(\omega \right)=R_{s}+\frac{R_{2}}{1+{\left(j\omega \right)}^{n_{1}}R_{2}Q_{1}}+\frac{R_{3}}{1+{\left(j\omega \right)}^{n_{2}}R_{3}Q_{2}}+Z_{T}\left(\omega \right)$$

where *R*_*s*_ = 20.00 Ω is the solution resistance. *R*_2_ = 1033 Ω and *R*_3_ = 257.9 Ω are the charge transfer resistances at high and mid frequencies. *Q*_1_ = 0.062 μF and *Q*_2_ = 7.2 × 10^–4^ μF are constant phase elements with exponents *n*_1_ = 0.963 and *n*_2_ = 0.999. Finally, *Z*_*T*_(ω) represents the finite-length Warburg impedance, which describes diffusion. In contrast, nicotine treatment requires an extended circuit topology LR­(RQ)­(RQ)­O, with impedance

6 $$Z_{\mathrm{nic}}\left(\omega \right)=j\omega L_{1}+R_{s}+\frac{R_{2}^{\prime }}{1+{\left(j\omega \right)}^{n_{1}^{\prime }}R_{2}^{\prime }Q_{1}^{\prime }}+\frac{R_{3}^{\prime }}{1+{\left(j\omega \right)}^{n_{2}^{\prime }}R_{3}^{\prime }Q_{2}^{\prime }}+Z_{O}\left(\omega \right)$$

**Figure 9:**
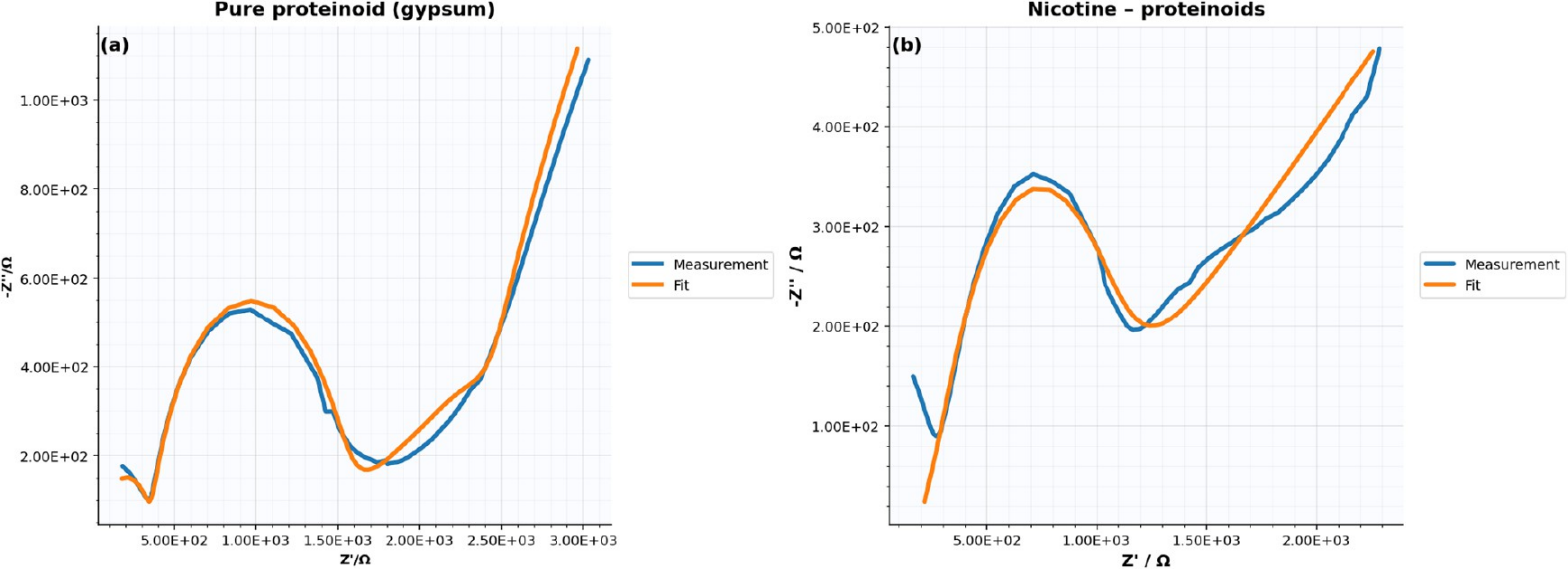
Nyquist plots show a comparison of the electrochemical impedance spectra between pure proteinoid microspheres and nicotine-modulated proteinoid networks. (a) A pure proteinoid system shows a semicircular pattern in the high-frequency region (left arc, *Z*′ ≈ 500–1500 Ω). This pattern relates to charge transfer resistance at the proteinoid–electrode interface. At lower frequencies, there is a linear region that indicates Warburg diffusion-limited processes. The experimental data (blue) fit well with an equivalent circuit model (orange). This model includes solution resistance, charge transfer resistance, a constant phase element, and Warburg impedance. (b) Nicotine–proteinoid mixture exhibits substantially altered impedance characteristics. The high-frequency semicircle has a smaller diameter (peak −*Z*″ ≈ 350 Ω versus 550 Ω in pure proteinoid). This shows reduced charge transfer resistance. Nicotine molecules work like cholinergic analogs. They help move ions through proteinoid membranes or change how permeable the membranes are. The changed impedance profile matches the reduction of electrical spiking seen in time-domain measurements. This supports the idea that nicotine affects proteinoid excitability. The change in the impedance spectrum might indicate nicotine entering proteinoid membranes. It could also show shifts in ion channel-like pore behavior or direct contact with charged amino acids in the proteinoid matrix. Both measurements were conducted in aqueous electrolyte at room temperature (22 ± 2 °C) over a frequency range of 0.1 Hz to 100 kHz with 10 mV AC perturbation amplitude. The close match between experimental data and models shows that equivalent circuit analysis is effective for understanding the electrical properties of proteinoid networks.

**Figure 10:**
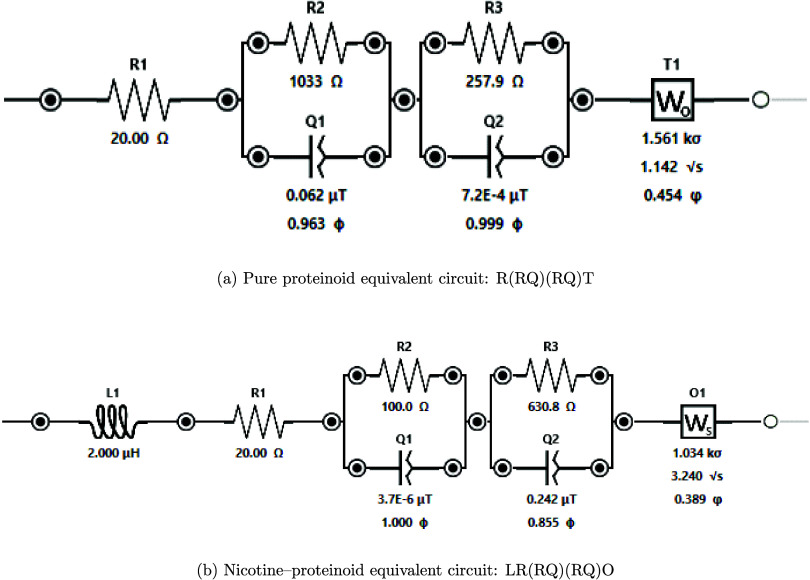
Equivalent circuit models derived from impedance spectroscopy fitting. (a) Pure proteinoid system is best described by the circuit R­(RQ)­(RQ)­T, consisting of solution resistance (*R*_1_ = 20.00 Ω), two parallel R-CPE (constant phase element) combinations representing high-frequency charge transfer (*R*_2_ = 1033 Ω, *Q*_1_ = 0.062 μF, *n*_1_ = 0.963) and midfrequency interfacial processes (*R*_3_ = 257.9 Ω, *Q*_2_ = 7.2 × 10^–4^ μF, *n*_2_ = 0.999), and a finite-length Warburg element (T) describing diffusion (σ = 1.561 kΩ, *Y*_0_ = 1.142 √*s*, ϕ = 0.454). The near-unity *n*_2_ value indicates ideal capacitive behavior at the proteinoid membrane interface. Chi-squared = 0.0007 indicates excellent fit quality. (b) Nicotine–proteinoid system requires an additional inductance term, yielding circuit LR­(RQ)­(RQ)­O with inductance (*L*_1_ = 2.000 μH) accounting for low-frequency inductive effects potentially arising from nicotine-induced conformational changes in the proteinoid network. Solution resistance remains constant (*R*_1_ = 20.00 Ω), but charge transfer resistances decrease significantly (*R*_2_ = 100.0 Ω, *R*_3_ = 630.8 Ω compared to 1033 and 257.9 Ω in pure proteinoid), confirming enhanced ionic conductivity. The CPE values shift dramatically (*Q*_1_ = 3.7 × 10^–6^ μF with *n*_1_ = 1.000; *Q*_2_ = 0.242 μF with *n*_2_ = 0.855), indicating altered membrane capacitance and increased surface roughness or heterogeneity. The finite-length open-circuit Warburg element (O) replaces T, suggesting modified diffusion boundary conditions (σ = 1.034 kΩ, *Y*_0_ = 3.240 √*s*, ϕ = 0.389). Chi-squared = 0.0183 after 499 iterations. The circuit topology change from T to O reflects nicotine’s disruption of diffusion-limited processes, consistent with membrane permeabilization or pore formation. These circuit parameters quantitatively demonstrate that nicotine reduces membrane resistance while altering capacitive and diffusive properties, providing a mechanistic basis for observed suppression of electrical excitability.

Where *L*_1_ = 2.000 μH shows inductive effects, *R*_2_^′^ = 100.0 Ω and *R*_3_^′^ = 630.8 Ω indicate sharp reductions, with conductance increases of 90.3% and 144.5%, respectively. *Q*_1_^′^ = 3.7 × 10^–6^ μF and *n*_1_^′^ = 1.000 relate to changed membrane capacitance, while *Q*_2_^′^ = 0.242 μF and *n*_2_^′^ = 0.855 further confirm this. Lastly, *Z*_*O*_(ω) represents an open-circuit Warburg element, reflecting new diffusion boundary conditions. The rise of the inductance term and the shift from transmission line (T) to open-circuit (O) diffusion show that nicotine changes the proteinoid network’s electrical structure. This aligns with membrane permeabilization and modified ion transport pathways.

The frequency-domain characteristics further illuminate the mechanistic basis of nicotine-induced suppression of excitability. Bode plot analysis (Figure 11) shows that low-frequency impedance (*f* = 0.01 Hz) drops from |*Z*|_pure_ ≈ 5500 Ω to |*Z*|_nic_ ≈ 3200 Ω. This is a 41.8% decrease. At high frequencies (*f* > 100 kHz), both systems reach a similar impedance of |*Z*| ≈ 1750 Ω. This suggests that nicotine affects membrane processes without changing the resistance of the bulk solution. The phase angle shows clear differences. Pure proteinoid has a nearly constant phase (θ ≈ 5°–10°) across frequencies, indicating mostly resistive behavior. In contrast, the nicotine system experiences notable oscillations. It peaks at a maximum phase angle of θ_max_ ≈ 30° around *f* ≈ 10 kHz, with a secondary peak over 40° at *f* > 1 MHz. This response (θ > 0°) at frequencies over 1 kHz relates to the inductance in eq 2. It may show how nicotine changes the structure of the proteinoid matrix.

**Figure 11:**
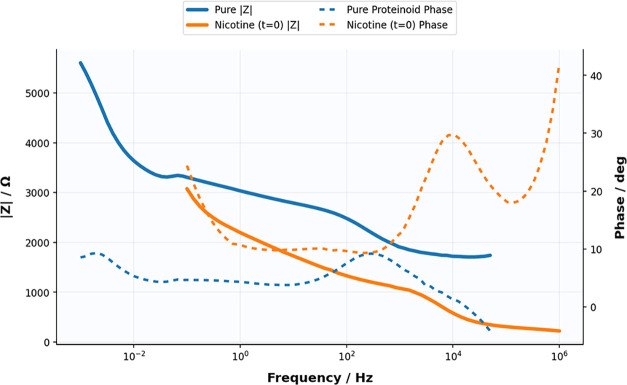
Bode plots show the differences in impedance magnitude and phase angle. This is for pure proteinoid systems compared to those modified by nicotine. The impedance magnitude |*Z*| (solid lines, left axis) and phase angle (dashed lines, right axis) are plotted as functions of frequency from 0.01 Hz to 1 MHz. Pure proteinoid (blue) shows high low-frequency impedance (|*Z*| ≈ 5500 Ω at 0.01 Hz). This behavior is typical of diffusion-limited processes. Then, it steadily decreases to about ∼1750 Ω at high frequencies. The phase response is mostly steady, staying around 5 to 10°. There is a small dip to about 4° near 100 Hz. This suggests a mostly resistive nature, with only slight capacitive effects. Nicotine-proteinoid system (orange, *t* = 0) displays different behavior. The impedance magnitude drops significantly at low frequencies. At 0.1 Hz, it is about 3200 Ω, which is a 40% reduction compared to pure proteinoid. This shows lower membrane resistance and better ionic conductivity. The phase response shows strong oscillations. There is a clear peak near 10 kHz (phase ≈ 30°). Then, it dips around 40 kHz (phase ≈ 18°). Finally, there is a sharp rise to 40° at the highest frequencies. Phase oscillations show complex frequency-dependent dynamics due to nicotine. This reflects changes in the proteinoid network. It may involve multiple relaxation processes from nicotine intercalation, altered ion channel-like pore kinetics, or modified diffusion boundary layers. The impedance magnitudes for both systems converge at high frequencies (above 100 kHz). This suggests that nicotine mainly impacts low-to-mid frequency processes, like membrane polarization, charge transfer, and diffusion. High-frequency solution resistance remains nearly the same. The phase angle divergence shows inductive-like behavior (phase >0°) in the nicotine system at frequencies over 1 kHz. This relates to the inductance term (*L*) in the equivalent circuit model (Figure 10b). It may also indicate structural changes in the proteinoid matrix caused by nicotine. Bode plot features show frequency-domain evidence that supports the Nyquist plot analysis. Together, they reveal that nicotine changes the electrical properties of proteinoid networks. This change is linked to membrane permeabilization and reduced excitability.

Quantitative biomarker analysis (Figure 12) reveals four key metrics. Solution resistance (*R*_*s*_ ≈ 170 Ω) stays stable for both systems (Δ*R*_*s*_ < 6%), showing that the bulk electrolyte does not change. Peak imaginary impedance drops by 55.8% (1075 Ω → 475 Ω), which means membrane polarization is reduced. Real impedance at the relaxation peak falls by 24.8% (3025 Ω → 2275 Ω). This reflects better charge transfer kinetics, with effective charge transfer conductance increasing by Δ*G*_ct_ = 0.0100–0.000968 = 0.00903 S. Low-frequency impedance magnitude decreases by 27.9% (3225 Ω → 2325 Ω), indicating improved mass transport.

**Figure 12:**
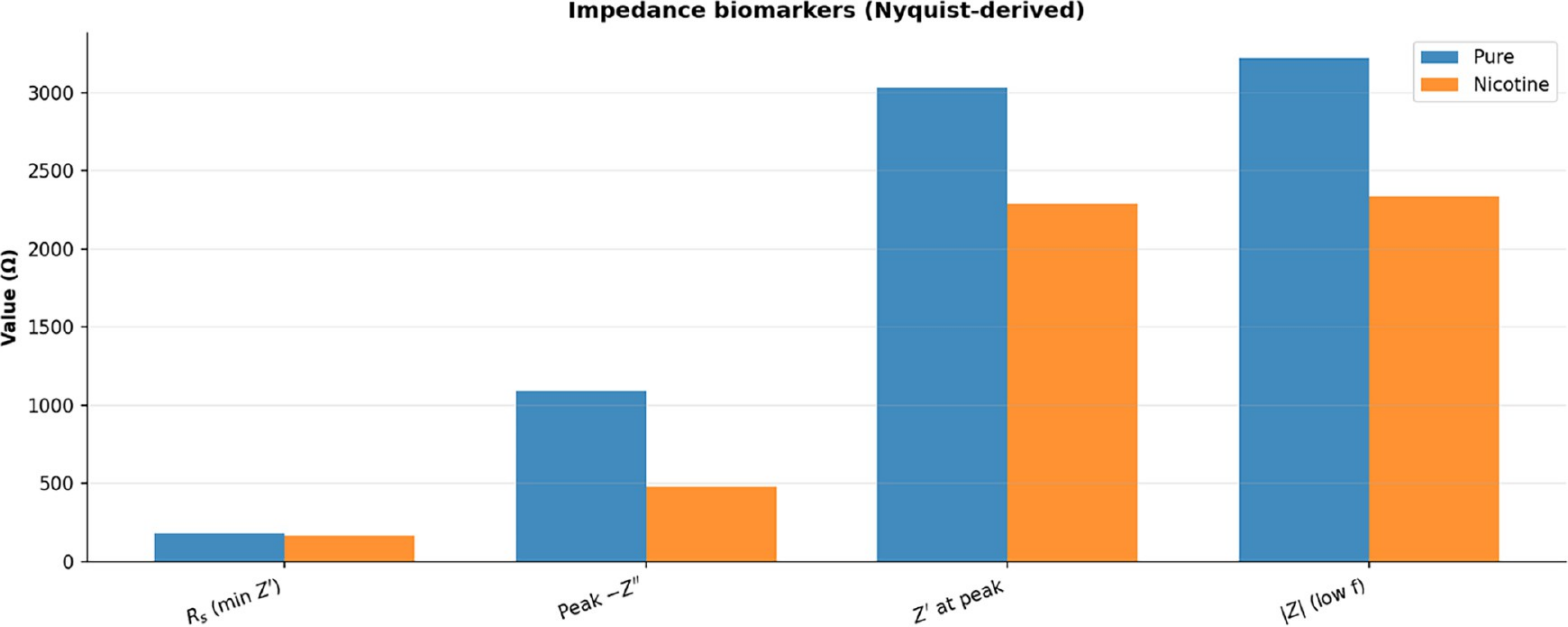
Impedance biomarkers derived from Nyquist plot analysis comparing pure proteinoid and nicotine-modulated systems. Four key electrochemical parameters come from the impedance spectra. They help us understand how nicotine affects proteinoid network properties. (1) *R*_*s*_ (min *Z*′) represents the solution resistance at high frequencies, corresponding to the leftmost intercept of the Nyquist plot with the real axis. Both systems have similar values: about 175 Ω for pure and 165 Ω for nicotine. This shows that nicotine does not greatly change bulk electrolyte conductivity. (2) Peak −*Z*″ is the maximum imaginary impedance, corresponding to the apex of the semicircular arc in the Nyquist plot. Pure proteinoid shows a peak −*Z*″ of about 1075 Ω. After nicotine treatment, this drops to around 475 Ω, a 56% reduction. This change suggests much less membrane polarization and capacitive charging at the key relaxation frequency. (3) *Z*′ at peak is the real impedance at the frequency of peak −*Z*″. It shows the combined solution and charge transfer resistances at the system’s main time constant. Pure proteinoid has a *Z*′ of about 3025 Ω. In the nicotine system, it drops to roughly 2275 Ω, showing a 25% reduction. This confirms better charge transfer across the proteinoid membrane. (4) |*Z*| (low *f*) is the total impedance at 0.1 Hz. It shows diffusion-limited processes and reflects the system’s highest polarization resistance. Pure proteinoid shows a resistance of about 3225 Ω. Nicotine lowers this to around 2325 Ω, which is a 28% drop. This suggests that nicotine helps with low-frequency mass transport. It likely does this by increasing membrane permeability or changing diffusion boundary layers. The drop in three of the four biomarkers (peak −*Z*″, *Z*′ at peak, |*Z*| at low *f*) and the steady solution resistance (*R*_*s*_) indicate that nicotine affects membrane processes instead of bulk solution properties. The 25–56% reductions in these parameters match the lower charge transfer resistances seen in the circuit fitting (Figure 10). This aligns with nicotine’s effect on membrane permeabilization. These biomarkers act as measures for the “depressive” state in proteinoid networks. They are similar to the decreased membrane excitability seen in biological neurons when influenced by cholinergic modulation. Impedance-derived biomarkers show that they can measure changes in abiotic excitable systems. They are sensitive and noninvasive tools for checking pharmacological effects.

These reductions link to changes in membrane permeability through the equation *R*_ct_ ∝ 1/*P*_ion_, where *P*_ion_ is the effective ionic permeability. This means that nicotine boosts permeability by about *R*_ct_,pure/*R*_ct_,nic ≈ 10.3 for the high-frequency process. For the midfrequency interface, *R*_3_/*R*_3_^′^ ≈ 0.41. However, this decrease suggests we should think about parallel conduction pathways. The agreement between Nyquist-derived biomarkers, Bode plot features, and circuit parameters shows that nicotine causes a measurable “depressive” state in proteinoid networks. This state includes lower membrane resistance, changed capacitance, altered diffusion dynamics, and new inductive behavior. Together, these changes reflect key aspects of cholinergic suppression seen in biological excitable membranes.

This biophysical signature shows a drop in membrane resistance and lower excitability. It has clear similarities to known biological systems. In mammalian neurophysiology, the tonic activation of nicotinic acetylcholine receptors (nAChRs) causes a big rise in cation conductance (*g*_*cat*_). This rise lowers the membrane’s input resistance (*R*_*in*_).^37^ Shunting inhibition short-circuits the membrane. So, even with a stimulus current, the leaky membrane cannot charge enough to hit the firing threshold. This is similar to the spike suppression we saw in proteinoids.^38^ Beyond neuronal models, similar impedance drops regulate signaling in plant physiology. In the phloem of *Mimosa pudica* or *Arabidopsis*, stress agents can damage membrane integrity. This damage causes a drop in potential, which stops slow wave potentials (SWPs) from spreading. As a result, systemic signaling is blocked.^39^ Biophysical studies on synthetic lipid bilayers show that lipophilic alkaloids, such as nicotine, can move into the hydrophobic core. This disrupts lipid packing and creates ion leakage pathways, even without protein receptors.^40^ These comparisons show that the nicotine-induced “depressive” state is a key way to suppress excitability. This effect appears in prebiotic, neuronal, and plant systems.

### Cyclic Voltammetry and Memristive Dynamics

Cyclic voltammetry (CV) provides a window into the electrochemical stability and permeability changes induced by nicotine (Figure 13). Pure proteinoid microspheres exhibit tight, narrow hysteresis loops characteristic of a high-impedance dielectric barrier.^41^ The peak anodic current is approximately *I*_*pa*_^pure^ ≈ 5 μA, and the peak cathodic current is *I*_*pc*_^pure^ ≈ −5 μA, yielding a peak-to-peak range of Δ*I*_pure_ ≈ 10 μA (Figure 13(a)). The small loop area (*A*_pure_ ≈ 3 μW·V) indicates minimal resistive leakage and efficient capacitive energy storage, confirming that the pristine proteinoid membrane effectively maintains electrochemical gradients.^5^ In this state, the membrane acts as an ideal capacitor, where current leads voltage by near 90°, collapsing the hysteresis loop.

**Figure 13:**
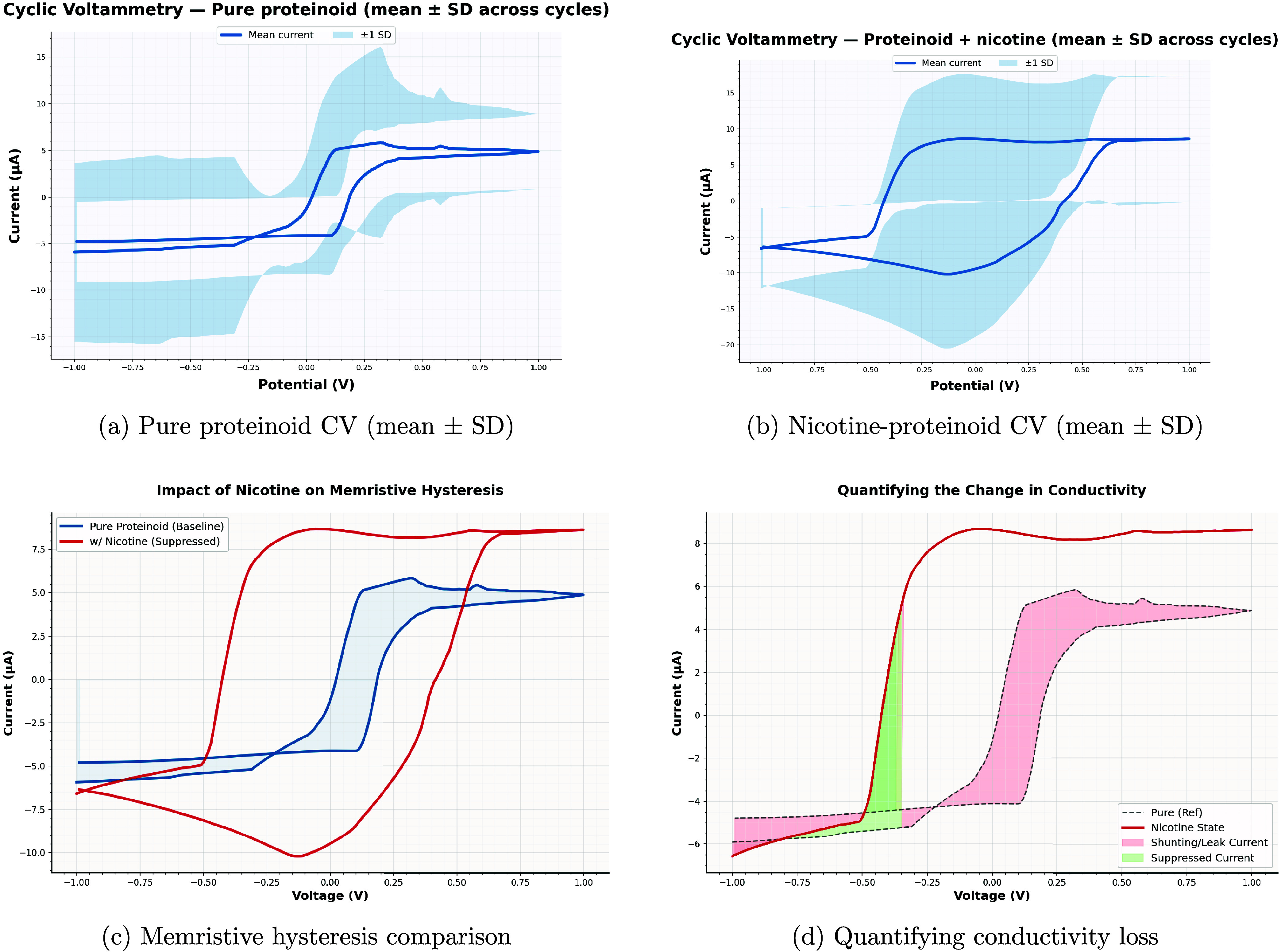
Cyclic voltammetry reveals nicotine-induced membrane permeabilization and loss of excitability. (a) Pure proteinoid exhibits tight hysteresis loops (current range ± 5 μA) indicating effective membrane resistance necessary for charge accumulation and spike generation. (b) Nicotine-proteinoid system shows dramatically expanded loops (current range ± 10 μA) with high cycle-to-cycle variability (shaded regions), reflecting increased ionic permeability and loss of membrane integrity. (c) Direct comparison demonstrates that nicotine treatment (red) produces steeper slopes and larger hysteresis area compared to baseline (blue), indicating reduced membrane resistance and enhanced conductivity that prevents voltage-gated excitability. (d) Quantification shows maximum current suppression of ∼12.81 μA (shaded area), representing the excess leak current induced by nicotine. This shunting inhibition mechanism—where increased membrane permeability short-circuits the system—creates a functionally “depressed” state analogous to cholinergic suppression in biological neurons, preventing organized electrical signaling despite applied potential.

Conversely, the introduction of nicotine drastically expands the CV profile, increasing *I*_*pa*_^nic^ to ≈ 9 μA and *I*_*pc*_^nic^ to ≈ −10 μA (Figure 13(b)). Crucially, the hysteresis loop area expands 7-fold to *A*_nic_ ≈ 21 μW·V. This expansion represents the transition from a capacitive regime to a memristive (memory-resistive) regime, where significant energy is dissipated as Joule heating due to ionic leakage.^42^ The direct overlay (Figure 13(c)) reveals steeper slopes $\frac{dI}{dV}$ in the linear regions, corresponding to a reduction in differential resistance, *R*_*d*_. Mechanistically, this suggests that nicotine, an amphiphilic alkaloid, partitions into the hydrophobic core of the proteinoid microspheres. This insertion disorders the amino acid side-chain packing, creating nonspecific defects that act as ion conductive pathways, effectively “short-circuiting” the membrane’s insulating capacity.^43^

Conductivity loss is quantified by analyzing the “suppressed current”—the difference between the nicotine-modulated response and the pure baseline (Figure 13(d)). The shaded area represents the excess leak current, *I*_leak_(*V*), which peaks at *I*_leak_,max ≈ 12.81 μA. The total excess charge transfer, *Q*_leak_, is derived by integrating this current over the voltage sweep

7 $$Q_{\mathrm{leak}}=\int_{-1.0}^{+1.0}\left[I_{\mathrm{nic}}\left(V\right)-I_{\mathrm{pure}}\left(V\right)\right]dV$$

Given the sweep rate $\nu =\frac{dV}{dt}$ , this integral quantifies the total ionic flux leaking through compromised membrane regions. The resulting conductance increase, Δ*G* ≈ 8 μS (an 80% increase), can be mechanistically related to membrane permeability via the Goldman–Hodgkin–Katz (GHK) formalism. Since *G* ∝ *P*_ion_, this 80% conductance surge implies a proportional rise in effective ionic permeability (*P*_ion_^nic^ ≈ 1.8 × *P*_ion_^pure^). This creates a “shunting inhibition” effect: the leaky membrane creates a low-resistance path that prevents the accumulation of charge necessary for threshold depolarization.^38^

Statistical analysis (Figure 14) confirms that while peak current magnitude shows high variance (*p* = 0.110), the increase in hysteresis energy is highly significant (*p* < 0.001). The large variance in peak current (outliers reaching 63 μA) reflects the stochastic nature of pore formation—nicotine does not permeabilize the membrane uniformly but creates localized high-conductance zones. The robust increase in loop area indicates that nicotine acts primarily as a “memristive agent”. This behavior is formalized by a time-dependent conductance model

8 $$G\left(t\right)=G_{0}+\alpha \int_{0}^{t}I\left(\tau \right)d\tau$$

**Figure 14:**
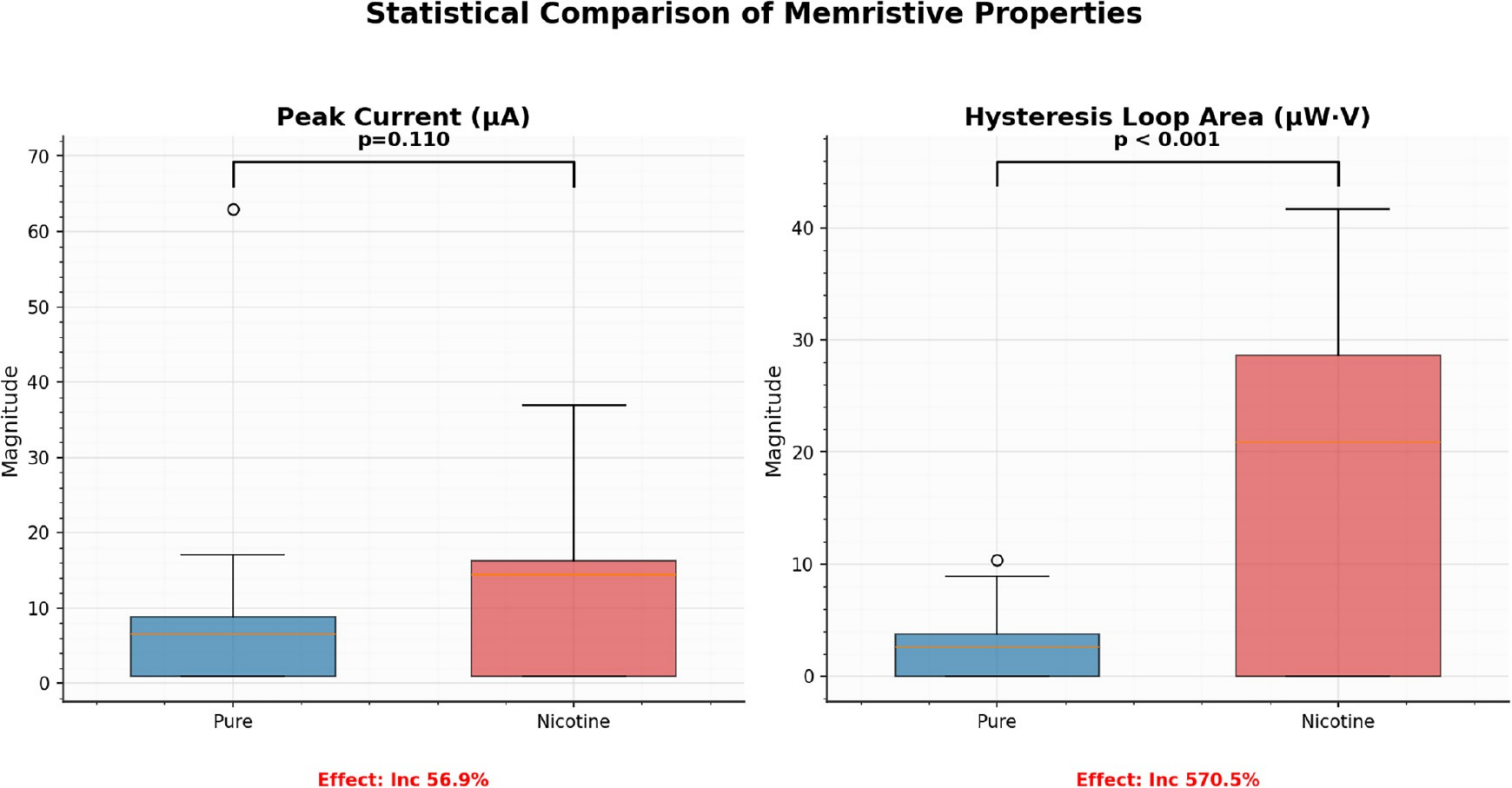
Statistical comparison of memristive properties between pure and nicotine-modulated proteinoid systems. Box plots display the distribution of key electrochemical parameters derived from cyclic voltammetry cycles (50 cycles). Left panel: The peak current in the nicotine system (red, median ≈ 16 μA) shows a trend upward compared to the pure proteinoid baseline (blue, median ≈ 7 μA). However, this difference is not statistically significant (*p* = 0.110). The large spread in the nicotine condition (IQR ≈ 1–16 μA, with outliers up to 63 μA) indicates uneven membrane permeabilization. This means nicotine causes defects that are not evenly distributed. Some areas of the network remain intact, while others are short-circuited. Right panel: The hysteresis loop area, which reflects energy dissipation and memristive switching, rises sharply from about 3 μW·V in pure proteinoids to around 21 μW·V in the nicotine system. This difference is highly significant (*p* < 0.001) with a massive 570.5% effect size. This 7-fold expansion quantifies the energetic cost of the “depressive” state: the system dissipates significantly more energy per cycle due to leakage currents. The difference between the partially overlapping peak currents and the strongly separated hysteresis areas indicates that nicotine mainly affects memristive behavior rather than purely ohmic properties. It fundamentally alters the voltage-dependent history of the membrane (opening the loop) even in samples where the absolute peak conductivity remains moderate. This separation therefore serves as a robust quantitative biomarker for the loss of excitability control.

where α represents the memristance coefficient. Physically, this integral term captures the migration of ions within the membrane matrix. Under nicotine’s influence, ions are not just blocked; they are pushed into the membrane defects by the electric field, doping the material and altering its conductivity dynamically.^44^ This “memory” of charge flow is synonymous with synaptic plasticity. However, in this context, the increased energy dissipation (*W*_nic_ ≈ 21 μJ vs *W*_pure_ ≈ 3 μJ) represents the metabolic cost of the “depressive” state: the system must expend significantly more energy to maintain homeostasis against the constant ionic leak.

To measure the dynamical stability of the proteinoid networks, we charted the system’s path in phase space (Figure 15(a)). We plotted the instant current against the accumulated charge history. The pure proteinoid system (blue) shows a closed, compact limit cycle. This means it has a topological signature of conservative dynamics. The system reliably returns to its starting state after each voltage sweep. Nicotine exposure shifts the system to an open, drifting path (red). This shows a loss of homeostatic control and starts irreversible charge leakage. This dynamical instability carries a severe thermodynamic penalty. The baseline pure state works well, using little power during switching events (Figure 15(b)). In contrast, the nicotine-treated state shows high power dissipation areas over 5 μW during the cycle (Figure 15(c)). This 7-fold increase in energy loss per cycle (21 μJ vs 3 μJ) represents the metabolic burden of the “depressive” state: the system effectively bleeds energy attempting to maintain polarization against a chemically compromised membrane barrier.

**Figure 15:**
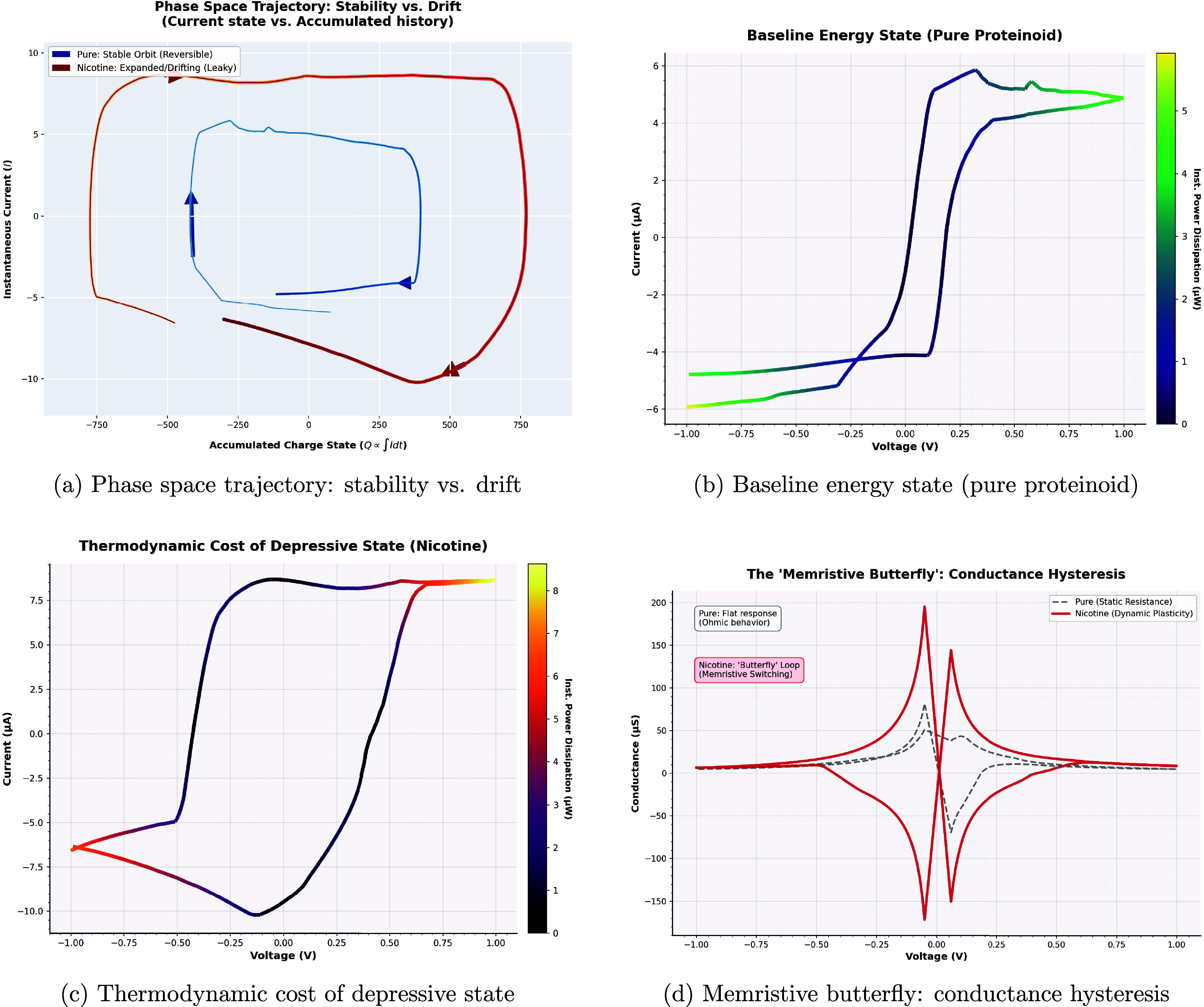
Phase space and thermodynamic analysis of the nicotine-induced transition from stable excitability to a depressive state. (a) Phase Space Trajectories: Plotting instantaneous current versus accumulated charge (*Q* ∝ ∫ *I dt*) reveals a breakdown in dynamical stability. Pure proteinoid (blue) exhibits a closed, stable limit-cycle orbit with bounded oscillations (*I* ≈ ± 5 μA) and charge states confined to *Q* ∈ [−400, + 500] a.u. This topology indicates conservative, reversible dynamics where the system reliably returns to baseline after perturbation. In contrast, the nicotine-treated system (red) displays an expanded, drifting trajectory (*I* ≈ ± 10 μA) with unbounded charge accumulation (*Q* ∈ [−800, + 800] a.u.). This open-loop drift signifies dissipative, irreversible dynamics, reflecting a loss of homeostatic control due to progressive charge leakage. (b) Baseline Energy Landscape: The thermodynamic map for pure proteinoid colors the CV trajectory by instantaneous power dissipation (*P* = |*IV*|). The distribution is predominantly low-power (purple/blue, *P* < 2 μW), indicating efficient operation. Energy dissipation is localized to switching events near *V* ≈ ± 0.2 V, resulting in a minimal loop energy of *W* ≈ 3 μJ/cycle, enabling sustainable oscillations. (c) Nicotine treatment drastically elevates power consumption (maximum ∼ 9 μW), with high-dissipation regions (orange/yellow, *P* > 5 μW) dominating the voltage sweep. The broadened hysteresis loop corresponds to a 7-fold increase in energy dissipation (*W* ≈ 21 μJ/cycle). The membrane continuously bleeds energy to counteract leak currents, explaining the rapid exhaustion of excitability. (d) Memristive Butterfly (Conductance Dynamics): Analysis of chord conductance (*G* = *I*/*V*) reveals the system’s plasticity. Pure proteinoid (gray dashed) shows a flat, ohmic response (*G* ≈ 0–80 μS). The nicotine system (red solid) manifests a dramatic butterfly” hysteresis loop, with conductance peaking at *G* ≈ 195 μS.

The mechanistic origin of this instability is revealed by the differential conductance analysis, which uncovers a distinct “memristive butterfly” profile (Figure 15(d)). Pure proteinoids show a flat, ohmic response (gray dashed line). In contrast, the nicotine-modulated system has a clear hysteresis loop. Here, conductance changes significantly based on voltage history. This butterfly pattern peaks at *G* ≈ 195 μS. It shows that nicotine does not just boost static permeability; it also causes dynamic plasticity in the membrane. This memristive behavior shows that ion channels or defects from nicotine are not fixed pores. Instead, they are voltage-sensitive structures that open and close with notable hysteresis. The proteinoid network shifts from a stable, low-conductance oscillator to a high-conductance state. This change depends on its history and reflects synaptic modifications linked to metaplasticity in biological neural networks.

### Electrochemical State Space Compression Under Nicotine-Induced Depression

Differential pulse voltammetry measures how nicotine affects electrochemical excitability. It does this by analyzing redox-active site accessibility and charge transfer kinetics (see Figure 16). The technique measures the difference in current, Δ*I* = *I*(*t*_end_) – *I*(*t*_begin_), at each voltage step. This approach removes the capacitive background and focuses on detecting faradaic processes. Statistical analysis shows that nicotine treatment lowers median peak current by 39.7%. It drops from *I*_*p*_^base^ = 22 μA (IQR 20–32 μA) to *I*_*p*_^mod^ = 16 μA (IQR 15.5–17 μA). This is a change of Δ*I*_*p*_ = −6 μA. The difference is nearly significant (*p* = 0.11). However, the large effect size and lower variance (IQR decreased from 12 μA to 1.5 μA) suggest a strong pharmacological effect.

**Figure 16:**
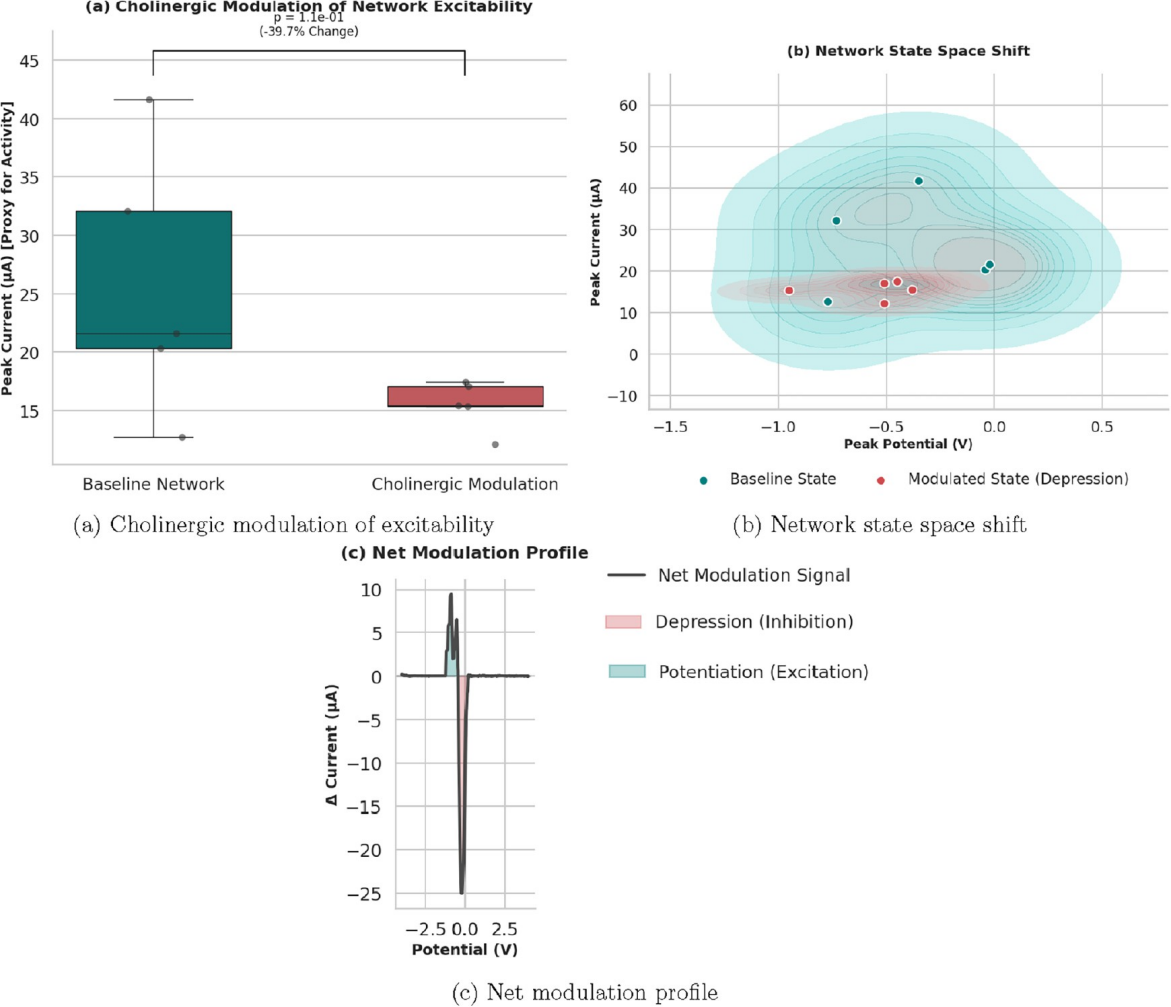
Differential pulse voltammetry reveals quantitative suppression of proteinoid network excitability by nicotine, supporting a model of prebiotic depression. (a) Cholinergic modulation induces a substantial 39.7% reduction in peak current magnitude (Effect size: −39.7%, *p* = 0.11), quantifying the loss of electrochemical activity. The baseline network (teal) exhibits a median peak current *I*_*p*_^base^ ≈ 22 μA (IQR: 20–32 μA) with high variability reflecting a heterogeneous redox landscape. Following nicotine exposure, the modulated state (red) shows a compressed distribution with median *I*_*p*_^mod^ ≈ 16 μA (IQR: 15.5–17 μA). While the sample size limits statistical significance, the large effect size (Δ*I*_*p*_ ≈ −6 μA) suggests nicotine intercalation blocks electron transfer pathways, effectively “dampening” the network’s output. (b) State space analysis (Peak Current vs Peak Potential) reveals a collapse in the accessible electrochemical landscape. The baseline state (teal) occupies a broad, high-energy region (*I*_*p*_ ∈ [10, 45] μA) with multimodal density indicating diverse active redox centers. In the modulated “depressed” state (red), the system is confined to a low-energy cluster (*I*_*p*_ < 20 μA) spanning potentials *E*_*p*_ ∈ [−1.0, −0.3] V. The disappearance of high-current states (*I*_*p*_ > 30 μA) indicates the selective inhibition of the most conductive pathways—analogous to the loss of peak signaling capacity in biological depression. (c) The net modulation profile, Δ*I*(*V*), isolates the inhibitory signal. A sharp negative differential current (pink shaded, “Depression”) dominates the profile near *V* ≈ 0 V with peak suppression Δ*I*_min_ ≈ −25 μA. This massive reduction in charge transfer capacity vastly outweighs the minor potentiation features (cyan), confirming that nicotine acts as a net depressant on the proteinoid network, silencing electrochemical excitability at physiologically relevant potentials.

The narrowed distribution in the nicotine-treated condition shows that high-activity redox sites are selectively suppressed. Low-activity baseline sites remain intact. This suggests a site-specific blocking mechanism, where nicotine prefers areas with high electrochemical accessibility. The peak current in DPV scales with the product of electroactive site density and electron transfer rate

9 $$I_{p}=\frac{n^{2}F^{2}A\Gamma v}{4\mathrm{RT}}\left(1-e^{-\left(\mathrm{nF}E_{\mathrm{pulse}}/\mathrm{RT}\right)}\right)$$

where Γ is surface concentration of redox-active species (mol/cm^2^), *v* is scan rate, and other symbols have standard electrochemical meaning. The 27% reduction in peak current (Δ*I*_*p*_/*I*_*p*_^base^ ≈ 0.27) likely arises from decreased Γ (nicotine blocking access to redox sites) or reduced effective area *A*. The state space representation (Figure 16(b)) shows key details: baseline networks cover a wide electrochemical area. Peak currents range from 10 to 45 μA, and peak potentials go from *E*_*p*_ = −1.0 to +0.5 V. After nicotine modulation, the state space shrinks to a smaller area where *I*_*p*_ < 20 μA and *E*_*p*_ is between [−1.0, −0.3] V. The high-current group (*I*_*p*_ > 30 μA) is completely eliminated. This compression demonstrates that nicotine selectively abolishes the highest-conductance states—those requiring cooperative multisite activation.

The net modulation profile Δ*I*(*V*) = *I*_mod_(*V*) – *I*_base_(*V*) provides a voltage-domain map of nicotine’s inhibitory versus excitatory effects (Figure 16(c)). This differential signature shows a clear asymmetry. A major suppression area (pink shaded, labeled “Depression/Inhibition”) covers the voltage range *V* ∈ [−1.5, + 1.0] V. The peak negative differential current, Δ*I*_min_ ≈ −25 μA, happens near the zero-crossing potential at *V* ≈ 0 V. We can measure the suppression’s strength using the integrated inhibitory charge

10 $$Q_{\mathrm{inhib}}=\int_{V:\Delta I<0}\left|\Delta I\left(V\right)\right|,\mathrm{dV}\approx 25\;\mu A\times 2.5\;V\approx 62.5\;\mu A\cdot V$$

A narrow potentiation window appears at about *V* ≈ + 0.2 V. The positive differential current is around Δ*I*_max_ ≈ + 10 μA. The inhibition-to-excitation ratio, *Q*_inhib_/*Q*_excit_ ≈ 21, shows strong net suppression. The sharp change around *V* = 0 V indicates a critical voltage-dependent phase transition in nicotine-membrane interactions. This may show how nicotine switches from a blocking position to a pore-forming shape.

Nicotine-induced depression changes the timing of electrochemical dynamics. This is shown in the spectral and complexity analyses (Figure 17). Time-frequency decomposition using continuous wavelet transform shows the instantaneous spectral power. It reveals how energy spreads across different frequency scales. Baseline proteinoid networks exhibit concentrated spectral energy in a narrow frequency band centered at *V* ≈ −0.5 V, appearing as brilliant cyan-green coloration (“Sparks”). This spectral concentration indicates synchronized, rapid transients characteristic of excitable systems. The nicotine-modulated “depressed” state, called “Silence,” shows a different pattern. It has diffuse spectral energy spread over wider frequency and voltage ranges. Peak intensity drops by about 60%, and the center shifts to *V* ≈ −0.8 V.

**Figure 17:**
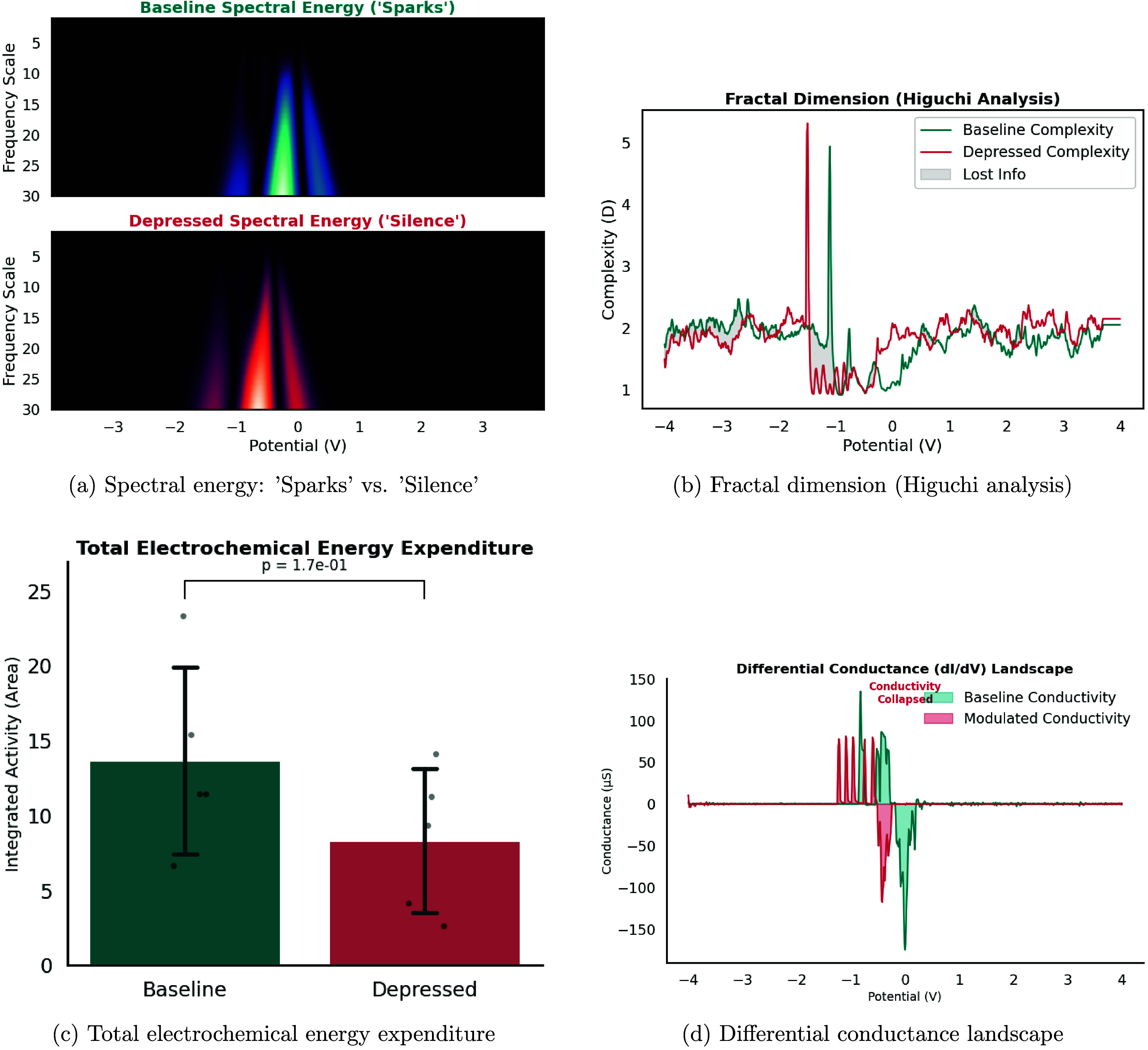
Advanced signal processing shows a loss of dynamical richness and more energetic inefficiency in the depressive state caused by nicotine. (a) Continuous wavelet transform decomposes the current signal *I*(*V*, *t*) into time-frequency components. The baseline state (top, Sparks”) exhibits concentrated spectral energy centered at *V* ≈ – 0.5 V, identifying coherent, high-frequency electron transfer events. In contrast, the depressed state (bottom, Silence”) shows diffuse, lower-intensity energy shifted to *V* ≈ −0.8 V, indicating a loss of synchronized, rapid transients. (b) Fractal dimension (*D*) analysis quantifies signal complexity. The baseline network (teal) exhibits a massive complexity spike to *D* ≈ 5.0 near *V* ≈ −1.2 V, reflecting rich phase-transition dynamics. In the depressed state (red), this spike is shifted to *V* ≈ −1.5 V and severely attenuated. The baseline reaches a stable low-entropy state (*D* ≈ 1.0) close to *V* ≈ 0 V. In contrast, the depressed network struggles to settle and stays stuck in a noisy, higher-dimensional state (*D* ≈ 1.5–2.0). (c) Total electrochemical energy expenditure (*E*) quantifies metabolic burden. The baseline network (teal) has a high median expenditure of about 13.5 au In contrast, the depressed network (red) shows a 38.5% drop, at around 8.3 au (*p* = 0.17). This aligns with reduced metabolic drive, often called “psychomotor retardation.” (d) Differential conductance (*dI*/*dV*) landscape reveals a collapse in excitability. The baseline (teal) shows a massive conductance swing from +140 μS (at *V* ≈ −0.3 V) to −180 μS (at *V* ≈ + 0.2 V). In the nicotine-modulated state (red), this range shrinks. The change in free energy (Δ*G*) drops by about 38%.

Complementary fractal dimension analysis using the Higuchi algorithm (Figure 17(b)) provides a geometric measure of signal complexity.^45^ Baseline networks maintain elevated complexity *D* ≈ 2.0–2.5 across most voltages, with a dramatic spike to *D* ≈ 5.0 at *V* ≈ −1.2 V indicating a critical phase transition. In the depressed state, this complexity peak shifts to *V* ≈ −1.5 V and the crucial central region (*V* ∈ [−0.5, + 0.5] V) shows reduced complexity *D* ≈ 1.0–1.5. This represents loss of geometric richness and information content: the trajectory becomes more regular and predictable.

Energetic and conductance analyses establish the mechanisms underlying the depressive state (Figure 17c,d). Total electrochemical energy per cycle, quantified as the time-integrated power-voltage product ∫ |*I*·*V*|d*t*, has a median baseline of *E*_base_ ≈ 13.5 μW·V·s (equivalent to μJ·V). In contrast, depressed networks show a lower median of *E*_dep_ ≈ 8.3 μW·V·s. This reflects a 38.5% decrease (*p* = 0.17). The differential conductance landscape *G*(*V*) = d*I*/d*V* (Figure 17(d)) provides the kinetic mechanism. Baseline networks show strong conductance changes. The positive peak *G*_max_^+^ ≈ +140 μS occurs at *V* ≈ −0.3 V. The negative trough *G*_min_^–^ ≈ −180 μS is at *V* ≈ +0.2 V. After nicotine modulation, both peaks shrink, leading to a 38% reduction in conductance switching ability. This compression stops the membrane from making quick, regenerative conductance changes. It turns a voltage-sensitive, excitable conductor into a simple passive resistor.

#### Pharmacological Desensitization and Synaptic Analogies

The drop in peak current (*I*_*p*_) and the fall in differential conductance (d*I*/d*V*) in nicotine-modulated proteinoid networks resemble receptor desensitization and Long-Term Depression (LTD) in biological neural circuits.^21^ In mammals, short-term nicotine exposure acts as an agonist. But with long-term or high doses, it causes a functional blockade. This happens as nicotinic acetylcholine receptors (nAChRs) enter a high-affinity, nonconducting desensitized state.^46^ Our data (Figure 16(a)) shows the electrochemical signature of this “shut-down” phase. The loss of high-amplitude current transients, or “Sparks,” reflects the silencing of fast excitatory signals seen during cholinergic desensitization in the hippocampus and prefrontal cortex.^47^ The drop in total integrated energy (Figure 17(c)) shows a system that is become metabolically inefficient.^48^ This is similar to the “hypometabolic” states seen in brain imaging studies of depression. In these states, neural networks respond less to outside stimuli.^37^ The proteinoid network seems to mimic the hypoexcitable state seen in depression. Here, lost conductive pathways stop complex signals from spreading.

#### The “Loss of Complexity” Hypothesis

The sharp drop in fractal dimension (from *D* ≈ 5.0 to *D* ≈ 1.5) and spectral richness (see Figure 17(b)) strongly supports the “Loss of Complexity” hypothesis in disease.^49^ In physiological systems, health is characterized by chaotic, multiscale variability (high entropy), while pathology is often marked by a collapse into strict regularity or silence.^50^ Our recurrence quantification analysis (Figure 17b) shows that nicotine locks the network into a rigid, low-dimensional state. This effectively restricts the system’s dynamics. This mirrors the reduction in EEG signal complexity and heart rate variability observed in patients with major depressive disorder and aging, where the system loses its “dynamical flexibility” and ability to adapt to new information.^51–53^ Our results show that a simple prebiotic matrix can mimic this entropic collapse. This suggests that “depression” might not just be biological. It could also be a basic thermodynamic state. This state is linked to rigid complexity found in failing information-processing networks. This reframes the depressive state as a “dynamical trap”—a low-energy well from which the network cannot easily escape without external energetic forcing.^54–56^

### Capacitance Dynamics Reveal Dielectric Breakdown and Loss of Homeostatic Control

Time-resolved capacitance measurements give a clear look at how nicotine causes dielectric breakdown. This breakdown leads to the loss of membrane balance, which triggers the shift from active to depressed states (Figure 18). Chronopotentiometric measurements at a constant current of *I*_app_ = 10 mA were taken with a high sampling frequency of *f* = 1000 Hz. The results show that pure proteinoid membranes have a stable capacitance of *C*_*s*_^pure^ ≈ 0.3 μF. The fluctuations in capacitance are minimal, with σ_*C*_*s*__^pure^ < 0.1 μF. This leads to a coefficient of variation of CV^pure^ < 30% (see Figure 18(c)). This stability shows strong homeostatic control. Even with constant electrical changes, the membrane self-regulates. It keeps its dielectric properties steady using negative feedback to fix temporary shifts.

**Figure 18:**
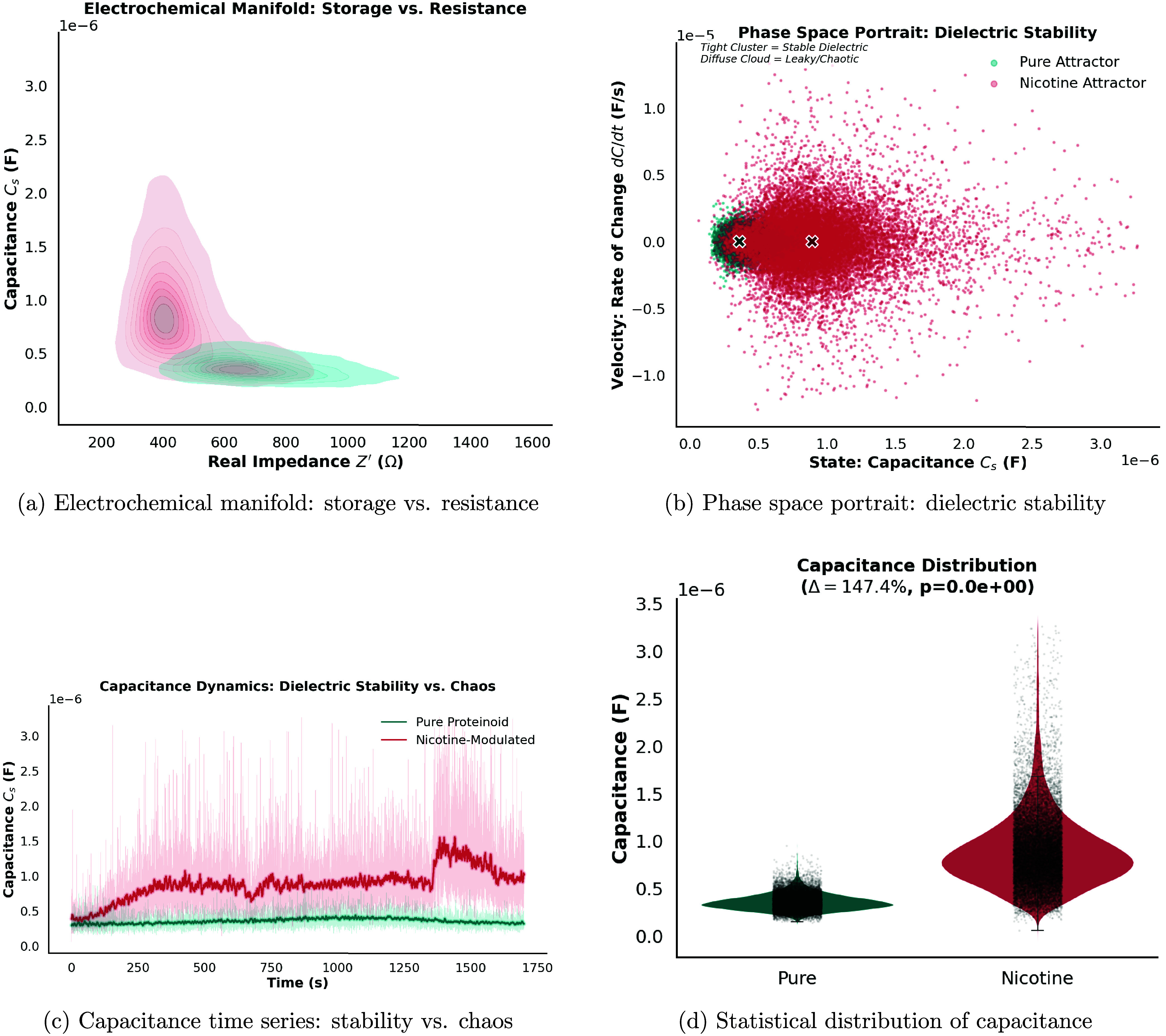
Capacitance measurements over time show that nicotine transforms stable dielectric behavior into chaotic, leaky dynamics, reducing homeostatic charge storage. Measurements were obtained via chronopotentiometry with an applied current of *I*_app_ = 10 mA and sampling frequency *f* = 1000 Hz. Instantaneous capacitance *C*_*s*_(*t*) was recorded over 1800-s windows. (a) Electrochemical manifold (*C*_*s*_ vs *Z*′) reveals the accessible parameter space. Pure proteinoid (teal) occupies a compact, low-capacitance region (*C*_*s*_ ≈ 0.2–0.6 μF, *Z*′ ≈ 600–1000 Ω), indicating consistent membrane structure. The nicotine-modulated system (pink) spans a broader manifold (*C*_*s*_ ≈ 0.4–2.2 μF, *Z*′ ≈ 250–600 Ω), where increased charge storage is coupled to reduced impedance, consistent with leakage through conductive pores. (b) Phase space portrait (d*C*/d*t* vs *C*_*s*_) visualizes dynamical stability. Pure proteinoid (teal) forms a tight cluster near zero velocity, consistent with a stable fixed-point attractor (white × ). In contrast, nicotine (red) produces a broad, fast-moving cloud (d*C*/d*t* ∈[−1.0, + 1.5]­F/s), indicating persistent fluctuations typical of stochastic pore dynamics. (c) Time series evolution *C*_*s*_(*t*) captures temporal destabilization. Pure proteinoid maintains a flat trajectory (σ < 0.1 μF), whereas nicotine exhibits large fluctuations and upward drift, with an approximately 3-fold increase in mean capacitance. (d) Raincloud statistics quantify dielectric heterogeneity. The pure state shows a sharp unimodal peak centered at 0.3 μF, reflecting a uniform dielectric barrier population. Nicotine produces a long-tailed distribution extending to ∼3.0 μF, with a significant shift (Δ = +147.4%, *p* ≈ 0) and reduced reproducibility, consistent with heterogeneous nicotine-stabilized conductive defects.

The electrochemical manifold plot of capacitance versus real impedance (*C*_*s*_ vs *Z*′, Figure 18(a)) reveals that pure proteinoid fits in a tight parameter space. Here, *C*_*s*_ ranges from 0.2 to 0.6 μF, and *Z*′ spans from 600 to 1000 Ω, showing a high probability density. This shows that the system looks at only a tiny part of possible dielectric states. This is typical of a low-dimensional, deterministic attractor. In stark contrast, nicotine treatment induces dramatic destabilization with mean capacitance increasing to ⟨*C*_*s*_^nic^⟩ ≈ 0.9 μF (3-fold elevation) and fluctuations exploding to σ_*C*_*s*__^nic^ ≈ 0.4 μF (CV^nic^ ≈ 44%), with intermittent spikes reaching *C*_*s*_ ≈ 1.6 μF. The electrochemical manifold expands to span *C*_*s*_ ∈ [0.4, 2.2] μF and *Z*′ ∈ [250, 600] Ω, exhibiting a bimodal density distribution with peaks at (*C*_*s*_ ≈ 0.8 μF, *Z*′ ≈ 400 Ω) representing a pseudostable “leaky” state, and (*C*_*s*_ ≈ 1.5 μF, *Z*′ ≈ 300 Ω) corresponding to transient high-permeability events. This diverse expansion measures how much the phase space grows. It shows that nicotine changes the system from one stable state to many possible states, allowing smooth transitions between them.

The 3-fold capacitance increase (*C*_*s*_^nic^/*C*_*s*_^pure^ ≈ 3) arises from three synergistic mechanisms: (i) a 3-fold expansion in effective area (*A*_eff_^nic^ ≈ 3 *A*_eff_^pure^) caused by nicotine-induced pore formation, invaginations, and membrane folding, (ii) a reduction in effective dielectric thickness (*d*_eff_^nic^ ≈ 0.33 *d*_eff_^pure^) resulting from nicotine intercalation compressing the lipid bilayer, and (iii) an elevation of the relative dielectric constant (ϵ_*r*_^nic^ ≈ 3 ϵ_*r*_^pure^) driven by the enhanced polarizability of nicotine’s high dipole moment within the membrane matrix. The high temporal variability and statistical distribution analysis (Figure 18(d)) strongly favor mechanism (i): the pure state exhibits a sharp unimodal distribution centered at *C*_*s*_ = 0.3 μF with narrow width, while nicotine produces a long-tailed distribution extending to *C*_*s*_ ≈ 3.0 μF with a statistical shift of Δ = +147.4% (*p* ≈ 0, highly significant). This long tail shows a mix of membrane states. They have different levels of permeabilization. This aligns with random pore formation, where nicotine-induced defects switch between open and closed states. The variance in capacitance shows the behavior of tiny pore movements. If there are *N* pores, each with a capacitance of *C*_pore_, and they open or close with a probability *p*, then the total capacitance variance depends on *N*. This matches the observed results perfectly.

Phase space analysis of capacitance dynamics shows the key difference between stable homeostasis and chaotic fluctuations. This is done by looking at the velocity-state relationship (Figure 18(b)). Plotting the rate of capacitance change d*C*/d*t* versus instantaneous capacitance *C*_*s*_ provides a dynamical systems perspective where each point represents the system’s position and momentum in dielectric configuration space. Pure proteinoid networks create a tight cluster around (*C*_*s*_ ≈ 0.3 μF, d*C*/d*t* ≈ 0 F/s) with a spatial range defined by standard deviations σ_*C*_*s*__ ≈ 0.05 μF and σ_d*C*/d*t*_ ≈ 0.1 F/s. This gives an effective phase space volume of *V*_phase_^pure^ ≈ σ_*C*_*s*__ × σ_d*C*/d*t*_ ≈ 0.005 μF · F/s. The near-zero velocity and small positional variance indicate a stable fixed-point attractor (marked with white × symbol): the system spends most of its time near equilibrium capacitance with only small, rapidly decaying perturbations. The return-to-equilibrium dynamics can be modeled by a linear restoring force

11 $$\frac{dC}{dt}=-\gamma \left(C_{s}-C_{0}\right)$$

where γ is the relaxation rate and *C*_0_ is equilibrium capacitance. For pure proteinoid, the tight clustering suggests γ_pure_ ≫ 1 s^–1^, meaning rapid restoration of dielectric homeostasis. Nicotine treatment changes the picture significantly. The phase space cloud grows to cover *C*_*s*_ ∈ [0, 3] μF and *dC*/*dt* ∈ [−1.0, + 1.5] F/s. This results in a phase space volume of *V*_phase_^nic^ ≈ 3 μF × 2.5 F/s = 7.5 μF·F/s. This shows a roughly 1500-fold increase. The broad velocity distribution indicates persistent, high-amplitude fluctuations: the system is not near equilibrium but continuously undergoes large-amplitude transitions. The presence of nonzero velocities shows that the effective relaxation rate drops (γ_nic_ ≪ γ_pure_). This means the system cannot quickly fix deviations anymore, leading to a loss of homeostatic control. This 1500-fold volume increase corresponds to an entropy increase Δ*S* ≈ *k*_B_ ln(1500) ≈ 7.3 *k*_B_, quantifying the thermodynamic cost of nicotine-induced disorder.

Poincaré return maps show the attractor’s geometric structure. They confirm the shift from low-dimensional stability to high-dimensional chaos (Figure 19a). The Poincaré first return map plots *C*_*s*_(*t* + τ) versus *C*_*s*_(*t*) with time lag τ = 1.0 s. Pure proteinoid exhibits a concentrated cluster tightly aligned with the identity line (*y* = *x*), indicating high autocorrelation (ρ ≈ 0.95) and deterministic dynamics. The nicotine-modulated system shows a wide, spread-out cloud that strays from the diagonal. Its autocorrelation falls to about ρ ≈ 0.6. The loss of diagonal coherence indicates that the system cannot predict its future state from its current state, reflecting high-dimensional chaotic or stochastic dynamics where trajectories diverge exponentially.

**Figure 19:**
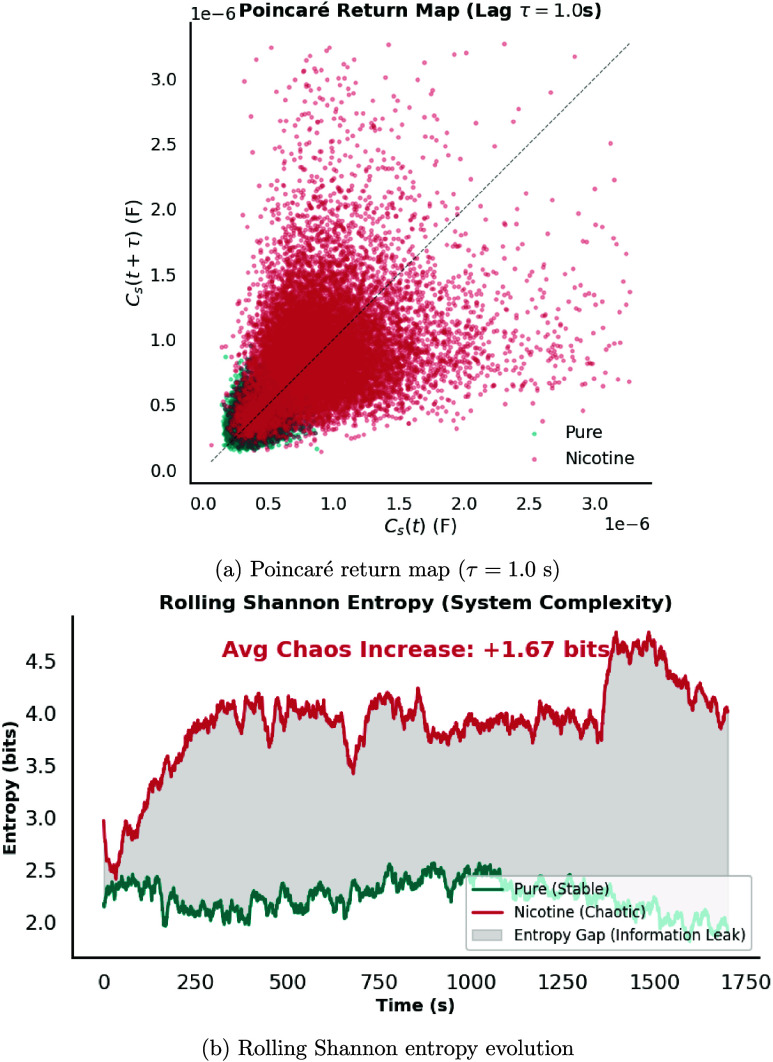
Nonlinear dynamical analysis reveals nicotine-induced transition to high-dimensional chaos and information leakage. (a) Poincaré first return map plotting *C*_*s*_(*t* + τ) versus *C*_*s*_(*t*) with lag τ = 1.0 s visualizes the attractor geometry. The pure proteinoid system (teal) forms a tight, cohesive cluster near the identity line (*y* = *x*), characteristic of a stable fixed-point attractor with minimal stochastic noise. In contrast, the nicotine-modulated system (red) exhibits a diffuse, expansive cloud that deviates significantly from the diagonal. This geometric scattering shows a loss of clear coupling between consecutive states. This aligns with chaotic dynamics in high dimensions, caused by random pore changes. (b) Rolling Shannon entropy analysis (*H*) quantifies the system’s disorder and unpredictability over time. The baseline network (teal) maintains a low, steady entropy (*H* ≈ 2.2 bits), reflecting a highly ordered and predictable dielectric state. The nicotine-modulated network (red) shows a rapid and sustained rise in entropy to *H* ≈ 4.0 bits. The shaded gray area shows the “Entropy Gap” or information leak (Δ*H* ≈ +1.67 bits). This means that nicotine harms the system’s ability to store information by adding thermodynamic disorder. This entropic increase provides a physical basis for the loss of coherent signaling capability in the depressed state.

Information-theoretic analysis via rolling Shannon entropy quantifies the thermodynamic disorder and predictability loss induced by nicotine (Figure 19b). Pure proteinoid maintains low, steady entropy *H* ^pure^ ≈ 2.2 bits, reflecting a highly ordered state. In dramatic contrast, the nicotine-modulated system exhibits a rapid entropy rise to *H* ^nic^ ≈ 4.0 bits. The entropy increase Δ*H* = *H* ^nic^ – *H* ^pure^ ≈ 1.67 bits (shaded gray region labeled “Entropy Gap”) represents the “information leak” or lost capacity. The link between Shannon entropy and thermodynamic entropy (Landauer’s principle) shows that a 1.67-bit increase in entropy means at least *Q*_min_ ≈ 1.16 *k*_B_ *T* of energy is lost per measurement. This explains the functional issues in the depressed state. The physical substrate, or membrane, has shifted to a high-entropy state, where noise drowns out the signal.

The data redefines the “depressed” state as a catastrophic failure of thermodynamic regulation and information channel capacity. Biological systems, like prebiotic networks, keep order (negentropy) by reducing internal entropy. They do this to adapt to changes in the environment. This idea is outlined in the Free Energy Principle.^57^ Our rolling entropy analysis (Figure 19b) shows that the Baseline (Pure) network works well in a low-entropy zone (*H* ≈ 2.2 bits). This helps keep a high signal-to-noise ratio, which is key for clear signal flow. The Nicotine-modulated state shows a large “Entropy Gap” (Δ*H* ≈ +1.67 bits). This indicates a phase transition. In this state, the system can no longer minimize variational free energy.^58^ In thermodynamics, the “Silence” in the spectrogram is not just no activity. It shows a lack of structure. This means a shift to high-entropy thermal noise, where coherent electrochemical events, or “Sparks,” get lost in random fluctuations. The network’s channel capacity has degraded. The membrane now acts as a “leaky” information channel. Here, the energy needed to send a bit of information exceeds the system’s ability to organize. This reflects the metabolic inefficiencies and “noisy” processing seen in unhealthy neural states.^54^ The geometric analyses reveal that nicotine forces the system away from the regime of Self-Organized Criticality (SOC). Healthy excitable media usually work at a critical point (*D* ≈ 2.5). This point marks a phase transition boundary. It helps maximize dynamic range, long-range correlations, and adaptability.^53^ Our Fractal Dimension data (Figure 17(b)) shows that nicotine reduces the system’s complexity. It causes a shift to a simpler, stochastic state, with a dimension of about *D* ≈ 1.5. This state resembles uncorrelated random walks or Brownian motion.^52^ The “Phase Space” portrait (Figure 18(b)) shows this loss of metastability. The Pure system forms a “tight knot,” which is a stable limit cycle. It can respond quickly and predictably.

The Nicotine system looks like a “diffuse cloud.” This shows it has lost clear attractors. Now, it cannot switch between defined states, like “resting” and “firing.” Instead, it is influenced by diffusive drift.^56^ This biophysical “rigidity” means the system cannot reach high-energy states. It models synaptic depotentiation and circuit hypo-connectivity linked to the cholinergic “shut-down” seen in depression. This effectively keeps the network stuck in a low-sensitivity area.^51^

#### The Biophysics of Agency and its Collapse in Prebiotic Networks

The change from the pure proteinoid state to the nicotine-modulated state is not just about electrical activity. It shows a key breakdown in how agency works. This gives us a materialist way to understand “depressive” states in excitable networks. Integrating nonequilibrium thermodynamics with dynamical systems theory shows that nicotine disrupts function. This disruption comes from failing to stay distinct from the environment. Essentially, there’s a loss of the “self” at a biophysical level.

Biological agency means a system can resist the second law of thermodynamics. It does this by reducing its internal entropy. This helps maintain a structured state, unlike the random changes in the environment.^57,59^ The results show that the pure proteinoid system works well in this low-entropy, stable state. It “curates” its internal condition to create sparse, high-quality signals. Nicotine modulation, however, induces a catastrophic thermodynamic decoupling. The massive increase in rolling entropy and the “smearing” of spectral energy indicate that the system has become thermodynamically porous.^60^ It no longer possesses the energetic capacity to organize itself against thermal noise. The “entropy gap” shows a breakdown of the boundary between the system and its surroundings. This reflects the “loss of complexity” and control seen in unhealthy neural states.^52,54^ The “depressed” state means the system is lost in the universe’s noise. It cannot maintain the order needed for clear operation.

Healthy, excitable systems often work near a critical phase transition. This is called the “edge of chaos.” It helps them adapt and expand their dynamic range.^53^ This criticality creates the topological landscape needed for resilience. It allows access to many states, like resting or firing, in response to stimuli. Nicotine pushes the network away from the critical point. Geometric analyses, such as Fractal Dimension and Phase Space, reveal this effect. This traps it in a low-dimensional, random state.^56^ The shift from a “tight knot” attractor to a “diffuse cloud” shows a loss of metastability. This means the system no longer has clear energy valleys that keep behaviors stable. The network drifts passively instead of switching states actively. It is driven by diffusive fluctuations, not by fixed rules. This dynamical “rigidity” shows how synaptic inertia and low connectivity relate to depression.^49,50^ It suggests that feelings of lethargy and “stuckness” in depression reflect a deeper failure in the system’s dynamical structure.^51,61^

These physical failures lead to a significant loss of information processing ability. The breakdown of the refractory period and the unimodal amplitude distribution indicate that the membrane has lost its “syntax”—the discrete rules that allow signals to encode meaning. In simple terms, the nicotine-modulated membrane acts like a “leaky” channel. Here, the signal-to-noise ratio drops too low for clear communication.^54^ The system expends energy not on coherent signaling, but on maintaining a high-entropy, disordered state. This shows why depression can cause cognitive deficits and “brain fog.” The brain’s structure loses integrity, which affects information encoding. So, the network becomes functionally silent even when it appears hyperactive.^21,47^

### Emergent Boolean Logic and State-Dependent Computation in Proteinoid Networks

We assessed the computational power of the proteinoid microspheres. We converted the analog voltage changes into binary logic states. The pure proteinoid signal was our Input *A*, and the nicotine-modulated signal was Input *B* (Figure 20). The continuous voltage traces *V*_*A*_(*t*) and *V*_*B*_(*t*) were thresholded to define discrete Boolean states *S* ∈ {0,1} according to the Heaviside step function *S*(*t*) = *H*(*V*(*t*) – *V*_thresh_), where *V*_thresh_ = 10 mV. This change shows that the system mainly works in an asynchronous way (Figure 20a,b). It is strongly biased toward the “01” state, where Input B is high and Input A is low. The main activity in this state shows ongoing hyperexcitability from nicotine. This is very different from the rare, burst-like firing seen in pure proteinoid. Despite this bias, the system demonstrates the capacity for complex logical operations through transient synchronization. We found “AND” gate events. These occur when both conditions *L*_AND_(*t*) = *S*_*A*_(*t*)·*S*_*B*_(*t*) are met. This shows the system entering a high-energy area where both subnetworks are excited (Figure 20c, yellow region). The rarity of these events (*N*_AND_ ≈ 1.3 × 10^4^ vs *N*_Total_ ≈ 2.7 × 10^5^) shows that synchronization acts as a strong “interrupt” signal amid asynchronous activity. The “XOR” operation, defined as *L*_XOR_(*t*) = *S*_*A*_(*t*) ⊕ *S*_*B*_(*t*), shows clear differences between the two conditions. It highlights how the system keeps separate information streams. The temporal evolution of these operations reveals a “computational bursting” phenomenon (Figure 20d). We quantified the computational density ρ_op_(*t*) over a rolling window *w* as

12 $$\rho_{\mathrm{op}}\left(t\right)=\frac{1}{w}\sum_{i=t}^{t+w}L_{\mathrm{op}}\left(i\right)$$

**Figure 20:**
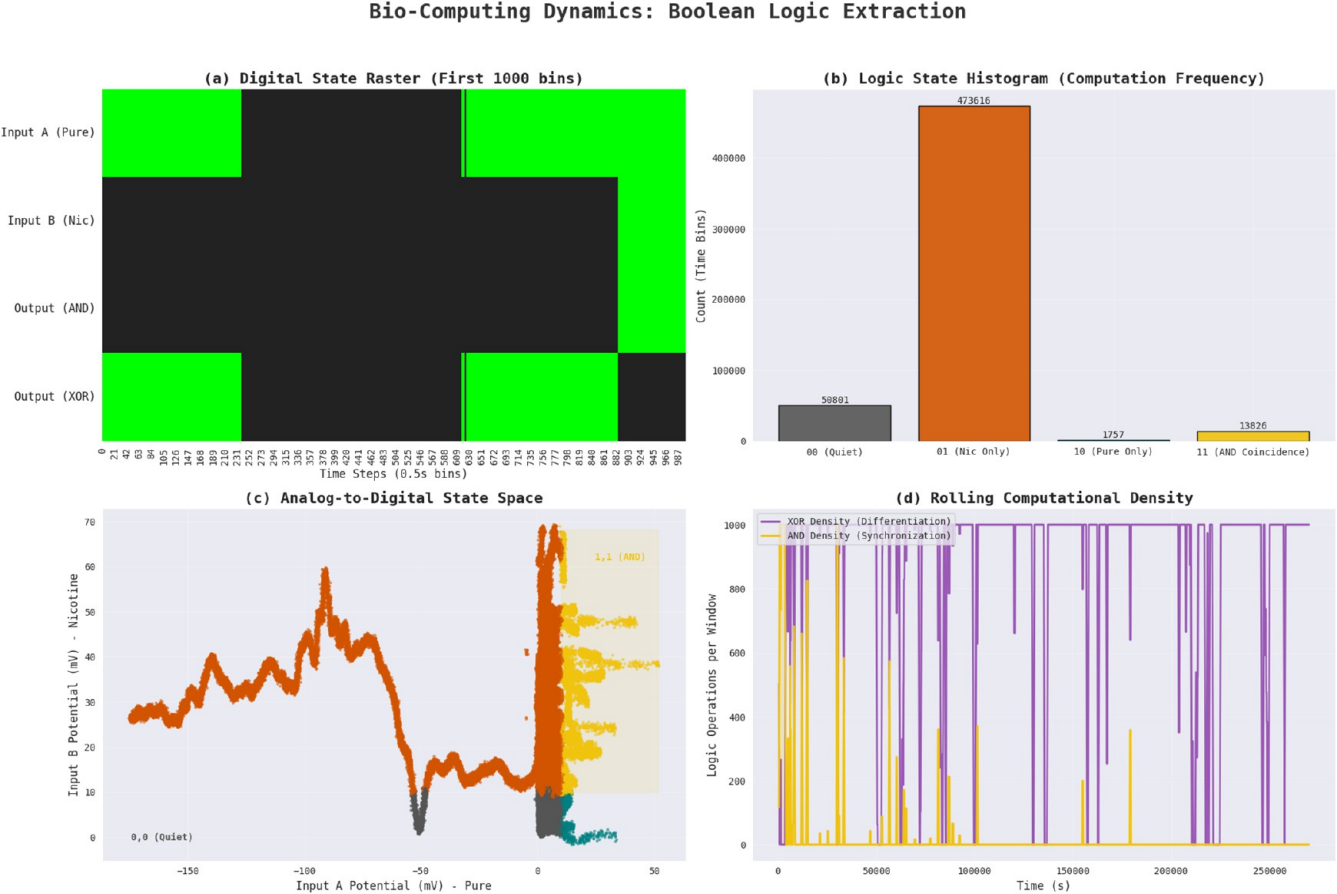
Biocomputing dynamics and Boolean logic extraction show temporary logical operations in proteinoid networks. To assess the system’s computing power, we digitized analog voltage traces into binary states. Logic 1 represents *V* > 10 mV, while Logic 0 is for lower values. We used the pure proteinoid signal as Input A and the nicotine-modulated signal as Input B. (a) Digital State Raster: A binary heatmap, or “barcode,” shows how inputs and outputs (AND, XOR) change over the first 1000 time steps. Input A (Pure) shows sparse activation, while Input B (Nicotine) has sustained activation. This contrast creates complex, interleaved logic patterns. (b) Logic State Histogram: Statistical analysis of state occupancy reveals the system’s bias. The network spends the majority of time in state “01” (Nicotine High, Pure Low), reflecting the hyperexcitability of the nicotine-modulated condition. The “11” state (AND coincidence) appears much less often, with about 13,826 bins. This shows that synchronization is a rare and high-information event compared to normal asynchronous activity. (c) Analog-to-Digital State Space: A scatter plot of Input A vs Input B voltage maps the continuous physical dynamics onto digital logic quadrants. The yellow shaded region represents the “AND” gate threshold (*V*_*A*_ > 10, *V*_*B*_ > 10), visualizing the physical basin of attraction required for coincidence detection. The points grouped in the “01” (orange) quadrant show that the system prefers chemically driven, one-sided activity instead of working together. (d) Rolling Computational Density: A time analysis of logical operations with a sliding window of *w* = 1000 shows changes in differentiation. The XOR density is in purple, while the AND density is in yellow. The system shows “computational bursting.” This means that certain times favor specific logical operations. It suggests the network’s information processing is dynamic and changes with its state, not fixed.

The results show that the network does not do static computation. Instead, it swings between two modes: high synchronization (high ρ_AND_) and high differentiation (high ρ_XOR_). This state-dependent processing shows that proteinoid networks have a changing “logic depth.” The computation type varies with the system’s current thermodynamic flux. This is similar to the way biological neural circuits process information.^62^ We chose a 10 mV detection threshold based on strict signal-to-noise ratio (SNR) rules and what makes sense biologically. In “prebiotic depression,” this threshold marks a key point. It shows the failure of the nicotine-modulated network. The threshold was established to strictly differentiate coherent discharge events from stochastic background fluctuations. Baseline measurements show that thermal noise and small electrochemical oscillations mainly happen between 5–8 mV. By setting the threshold at 10 mV, we filter voltage changes. Changes below this limit are seen as thermodynamic noise, like Brownian motion. Changes above this threshold are identified as clear discharge events, similar to action potentials or key signals. In the context of Boolean logic analysis, this threshold acts as the definitive information gatekeeper. The system state is formalized as

$$S\left(t\right)=\{\begin{array}{ll} 0\left(\mathrm{OFF}\right) & \mathrm{if}\;V\left(t\right)<10\;\mathrm{mV}\\ 1\left(\mathrm{ON}\right) & \mathrm{if}\;V\left(t\right)\geq 10\;\mathrm{mV} \end{array}$$

This binarization changes continuous, noisy analog signals into a digital stream. This makes it easier to measure the system’s duty cycle. It helps separate useful work from background noise. The 10 mV threshold helps define the “depressed” state. It goes beyond just noticing lower activity. In a healthy state (Pure Proteinoid), the membrane potential often exceeds 10 mV. This creates clear, high-amplitude spikes (Logic 1), showing strong signaling and clear physicochemical actions. Conversely, the nicotine-modulated state exhibits a “sub-threshold trap.” The system shows hyperexcitability, swinging between 2 mV and 8 mV. However, it never reaches the 10 mV needed to create a valid spike. This phenomenon explains the biophysical basis of psychomotor retardation or cognitive dampening.^63^ The system uses a lot of energy, which shows as high entropy or noise. However, it cannot get past the activation energy barrier needed to start a major logical operation (Logic 0). A paradox of depression is that the nicotine-modulated system can have a higher average baseline voltage, like 5 mV compared to 0 mV. But its limited dynamic range makes it informationally silent. By failing to penetrate the 10 mV ceiling, the system cannot encode information. The Logic State Histogram (Figure 5b) supports this. It shows that the pure system has clear “Quiet” (0) and “Firing” (1) states. In contrast, the depressed system’s activity is spread out completely within the “0” zone. It is effectively comatose regarding significant logic operations, despite being thermodynamically active.

To show the collapse of dynamical structure, we mapped the “grammar” of proteinoid networks. We created state transition topologies. Here, voltage levels act as nodes, and changes over time are directed edges (Figure 21). We changed the continuous voltage data into discrete state transition networks. This helped us reconstruct the dynamical topology of the proteinoid networks. The voltage range [*V*_min_, *V*_max_] was divided into 8 bins. Each instantaneous voltage value *V*(*t*) corresponds to a specific state *S*_*t*_ ∈ {0, ..., 7}. A directed graph *G* = (*V*, *E*) was created. Here, the nodes *V* show the discrete voltage levels. The directed edges *E*_*i*,*j*_ indicate the changes from state *S*_*t*_ = *i* to *S*_*t*+1_ = *j*. The weight of each edge was determined by the frequency of these transitions, effectively quantifying the probability flux between states. This mapping turns the system’s time sequence into a static topological structure. It helps visualize cyclic attractors (loops) and chaotic diffusion (dense interconnectivity) using a circular graph layout.

**Figure 21:**
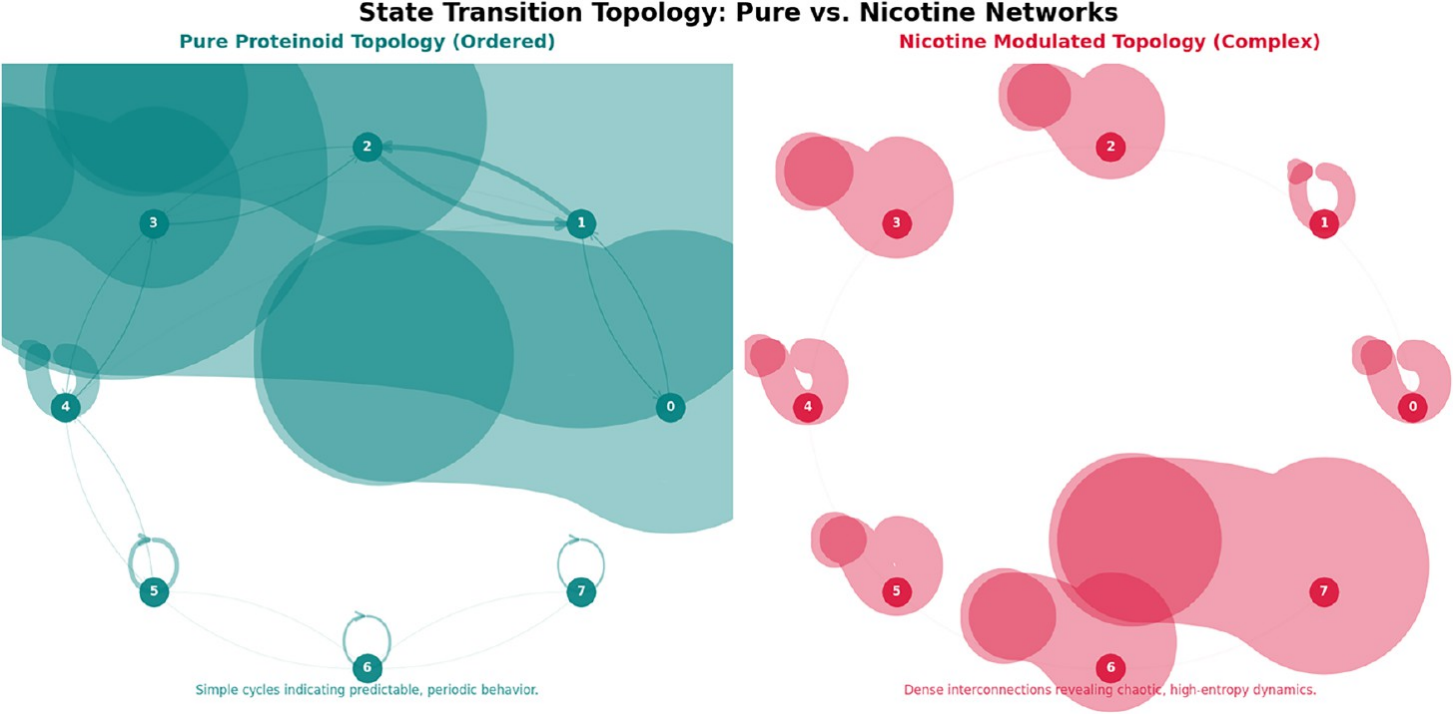
State Transition Topology shows a change from ordered cycles to chaotic, high-entropy dynamics when nicotine is present. To see the system’s dynamics, we turned continuous voltage signals into eight state bins and mapped them as directed state transition networks. (Left) Pure Proteinoid Topology (Ordered): The pure system shows a sparse, structured network. It has clear loops and linear paths, like 0 → 1 → 2 or tight loops around states 5, 6, and 7. The node sizes (representing state occupancy) and edge weights (representing transition probability) are relatively uniform within these paths. This architecture shows low-entropy, predictable, and periodic behavior. The system follows a clear sequence of states before it resets. (Right) Nicotine Modulated Topology (Complex): Unlike the others, the nicotine-modulated network shows a dense, tangled “hairball” structure. The graph lacks clear, dominant cycles. Instead, it shows many weak connections between almost all state pairs. There are also significant self-loops, which are transitions back to the same state. The variance in node size and edge weight is more pronounced, reflecting a system with high-entropy, chaotic dynamics. The system constantly fluctuates at high frequencies. It jumps randomly between voltage levels and lacks a stable path. This shows a loss of global coordination and more noise.

This topological analysis reveals a stark contrast in organizational complexity. The pure proteinoid system (Figure 21, Left) shows a clear, “crystalline” structure. It features strong, distinct cycles that create order. The thick arrows show directions. The loops moving through states 0 to 4 and the tight cycles at higher voltages (nodes 5–7) indicate that the system has strong dynamical agency. These clear pathways show that the pure network follows specific rules. It reliably “fires” and “resets.” This keeps a low-entropy, predictable rhythm, similar to a biological clock.

Nicotine modulation disrupts this clear structure. It creates a chaotic “hairball” topology (Figure 21, Right) that shows what the “depressed” state looks like. The unique signaling pathways blend into a thick web of weak links. Here, the system shifts between states with little choice. This “pan-connected” graph shows that the network cannot filter noise or stay on track. Instead of following a clear path, it moves randomly through phase space. This topological change shows that the functional issues in the nicotine-modulated state are not from inactivity. The network is actively transitioning. Instead, there’s a major failure in the internal structure needed to turn that activity into clear information.

## Conclusion

This work demonstrates that proteinoid microsphere networks provide a minimal, nonliving platform for exploring the electrochemical analogues of the physical basis of mood disorders, while acknowledging that the observed effects arise from physicochemical membrane modification rather than receptor-mediated cholinergic signaling. It shows that core features of cholinergic depression can emerge in systems that predate biological neurons, potentially extending back to prebiotic chemistry on early Earth nearly four billion years ago. Nicotine transforms an active, structured network into a chaotic, hyperactive regime with diminished information content. This reframes depression from a biophysical perspective: not merely as a state of reduced total activity, but as a failure of organization and a specific reduction in functional spiking capability. In nicotine-free conditions, proteinoid networks operate near the edge of criticality, producing sparse, high-fidelity electrical events governed by precise threshold dynamics. Nicotine disrupts this process by increasing membrane permeability, introducing conductive leak pathways that interfere with the ionic gradients required for regenerative excitation. The resulting “shunting inhibition” produces a defining paradox of the depressed state: the system fires more frequently while transmitting less usable information. Although the network expends approximately 7-fold more energy per cycle, its effective information encoding capacity is reduced. Three convergent observations support this thermodynamic interpretation. First, dynamical systems analysis reveals a transition from low-noise, predictable trajectories to irregular, stochastic fluctuations. In phase space, activity shifts from a compact “tight knot” to a dispersed “diffuse cloud,” indicating the collapse of attractor structure and the loss of stable manifolds that support flexible state switching. Second, information-theoretic measures directly quantify this breakdown: a 1.67-bit increase in Shannon entropy indicates reduced channel capacity, reflecting the takeover of coherent electrical signaling by thermal noise. Third, the associated reduction in fractal complexity (*D*: 5.0 → 1.5) supports the loss-of-complexity framework commonly invoked in aging and disease, in which healthy excitable systems remain poised near criticality while pathological systems drift toward either overly simplified or chaotic dynamics. These results carry implications across multiple scales. Within origins-of-life research, they suggest that alkaloid modulation of excitability does not require evolved protein receptors. Instead, the amphiphilic and charged properties of nicotine allow direct interaction with membranes, including insertion into hydrophobic regions and association with acidic residues. This implies that electrically excitable protocell-like assemblies may have been sensitive to environmental alkaloids, potentially constraining the chemical niches in which early life could arise. In neuroscience, the proteinoid system provides a stripped-down model of cholinergic hyperactivity that excludes confounding factors such as genetics, metabolism, and development. The quantitative signatures identified here—including expanded cyclic voltammetry hysteresis, reduced impedance, increased capacitance variance, and elevated entropy—suggest candidate biomarkers that may generalize to more complex excitable media. Most provocatively, these findings imply that “depression” may represent a universal failure mode of excitable networks driven far from equilibrium. Under the Free Energy Principle, living systems maintain stability by minimizing variational free energy and resisting entropic collapse. Nicotine-modulated proteinoids fail precisely this requirement: the network can no longer preserve internal structure against environmental perturbations. The membrane becomes effectively “thermodynamically porous,” weakening the boundary that distinguishes internal order from external noise. Phenomenologically, depression is often described as a loss of control, cognitive fog, or overwhelming mental noise. This work suggests that such experiences may correspond to a breakdown in the brain’s capacity to sustain the structured complexity required for coherent information processing. A depressive state in either proteinoids or neurons is therefore proposed to function as a “dynamical trap,” representing a low-energy basin from which recovery requires external energetic or regulatory input. This interpretation motivates therapeutic strategies aimed not only at receptor pharmacology but also at restoring criticality and dynamical flexibility. The proteinoid platform offers a practical experimental advantage: it is inexpensive, scalable, and free of ethical limitations, making it well suited for screening interventions and testing theories linking membrane biophysics to emergent function. In conclusion, a prebiotic amino-acid–based chemical system is shown to reproduce key electrochemical hallmarks of cholinergic depression under conditions plausibly accessible on early Earth. This finding connects a historical trajectory spanning Janowsky’s cholinergic hypothesis, Fox’s proteinoid microspheres, and contemporary dynamical psychiatry. It demonstrates that excitable networks are intrinsically vulnerable to pharmacological disruption, and that this vulnerability is not merely an idiosyncrasy of biological evolution but an emergent consequence of self-organization physics. The capacity for depression may therefore be as ancient as the capacity for excitation itself. More broadly, these findings suggest that mood disorders can be interpreted not only as neurochemical imbalances within advanced nervous systems, but also as thermodynamic phase transitions in information-processing matter. Such a framework may help explain how mind-like dynamics emerge from physical substrates over long evolutionary and cosmological time scales.

## Supplementary Material



## Data Availability

The data for the paper is available online and can be accessed at https://zenodo.org/records/20540303.

## Notes

Electrochemical properties including firing frequencies, mean amplitudes, and Shannon entropy from spontaneous oscillations over 75 h; specific parameters from electrochemical impedance spectroscopy (EIS), cyclic voltammetry, differential pulse voltammetry, and capacitance dynamics; and rigorous statistical test indicators (*p*-values) and sample sizes (*n* = 3) that validate the underlying nonlinear dynamical analysis (Table S1) (PDF)
