# Supplementary material for: Cholinergic Modulation of Proteinoid Microsphere Networks as Prebiotic Depression Models
Source: ACS Phys Chem Au. 2026 Jun 13;6(4):901–28. doi: 10.1021/acsphyschemau.6c00066 (PMC13397448; doi:10.1021/acsphyschemau.6c00066)
Supplement: Supplementary file 1 [file pg6c00066_si_001.pdf]

Supplementary Information: Cholinergic Modulation of Proteinoid Microsphere Networks as Prebiotic Depression Models

Panagiotis Mougkogiannis* and Andrew Adamatzky

Unconventional Computing Laboratory, University of the West of England, Bristol, UK

E-mail: Panagiotis.Mougkogiannis@uwe.ac.uk

Statistical Summary of Major Quantitative Claims

All electrochemical experiments were performed on $n = 3$ independently synthesized batches of proteinoid microspheres. Reported values are mean ± SD. Statistical comparisons used two-tailed Student's t-tests ($\alpha = 0.05$).

Notes: [a]NS: $p > 0.05$ but consistent large effect size across all replicates. [b]Circuit parameters from ZView non-linear least-squares fitting, mean ± SD across $n = 3$ fits. [c]$V_{\text{phase}} \approx \sigma_{C_s} \times \sigma_{dC/dt}$. [d]Rolling window 500 samples, 10 bins, $t > 5000\,\text{s}$. [e]Directly measured from replicate recordings; error = SEM across $n = 3$.

Table 1: Quantitative claims, replicates, and statistical parameters ($n = 3$; NS = not significant; n.a. = not applicable).

Parameter	Pure	Nicotine	Change	n	Test	p	Fig.
Spontaneous Oscillations (75 h)							
Firing freq.[e] (Hz)	$(3.02 \pm 0.35) \times 10^{-4}$	$(7.96 \pm 0.54) \times 10^{-4}$	+163.9 ± 35.2%	3	t	–	3
Peak count[e] N	76 ± 9	215 ± 15	+182.9 ± 37.8%	3	t	–	3
Mean ISI[e] (s)	3301 ± 415	1258 ± 24	−61.9 ± 4.8%	3	t	–	3c
Mean amplitude[e] (mV)	10.9 ± 0.9	25.5 ± 0.8	+133.9 ± 20.5%	3	t	–	3d
Phase space vol.[c]	0.005 ± 0.002	7.5 ± 1.8	×1500	3	t	<0.01	9b
Shannon entropy[d] (bits)	2.2 ± 0.3	4.0 ± 0.4	+1.67 bits	3	t	0.02	10b
Electrochemical Impedance Spectroscopy							
R_2 (Ω)[b]	1033 ± 95	100 ± 18	−90.3%	3	t	<0.001	5
R_3 (Ω)[b]	257.9 ± 32	630.8 ± 88	+144.5%	3	t	0.02	5
$-Z''_{\max}$ (Ω)	1075 ± 120	475 ± 65	−55.8%	3	t	0.01	6d
$\|Z\|$ 0.1 Hz (Ω)	3225 ± 310	2325 ± 280	−27.9%	3	t	0.03	6d
R_s (Ω)	175 ± 12	165 ± 14	−5.7%	3	t	0.61 NS[a]	6d
Cyclic Voltammetry							
Hysteresis area (μW·V)	3.0 ± 0.6	21.0 ± 2.8	+570%	3×20	t	<0.001	8e
I_{pa} (μA)	7 ± 4	16 ± 9	+129%	3×20	t	0.11 NS[a]	8e
ΔG (μS)	n.a.	8 ± 1.5	+80%	3	n.a.	n.a.	7d
Differential Pulse Voltammetry							
Median I_p (μA)	22 (IQR 20–32)	16 (IQR 15–17)	−39.7%	3	t	0.11 NS[a]	12a
Total energy (a.u.)	13.5 ± 2.1	8.3 ± 1.8	−38.5%	3	t	0.17 NS[a]	13c
Capacitance Dynamics							
$\langle C_s \rangle$ (μF)	0.3 ± 0.05	0.9 ± 0.18	+200%	3	t	<0.01	9c
Capacitance CV (%)	<30	≈44	+47%	3	–	n.a.	9d
Nonlinear Dynamical Analysis							
Fractal dim. D	5.0 ± 0.4	1.5 ± 0.3	−70%	3	t	0.01	13b
Poincaré ρ	0.95 ± 0.03	0.60 ± 0.07	−37%	3	t	0.02	10a
